# Identification of tumor-associated antigens and immune subtypes of lower-grade glioma and glioblastoma for mRNA vaccine development

**DOI:** 10.1186/s41016-022-00301-4

**Published:** 2022-10-28

**Authors:** Zhi-liang Wang, Ruo-yu Huang, Bo Han, Fan Wu, Zhi-yan Sun, Guan-zhang Li, Wei Zhang, Zheng Zhao, Xing Liu

**Affiliations:** 1 Department of Neurosurgery, Beijing Tiantan Hospital, Capital Medical University, Beijing, People’s Republic of China; 2 Department of Molecular Neuropathology, Beijing Neurosurgical Institute, Capital Medical University, No. 119 South 4th Ring West Road, Beijing, 100070 People’s Republic of China; 3 Department of Neuropathology, Beijing Neurosurgical Institute, Capital Medical University, No. 119 South 4th Ring West Road, Beijing, 100070 People’s Republic of China

**Keywords:** Glioma, mRNA vaccines, Antigens, Prognosis

## Abstract

**Background:**

mRNA became a promising therapeutic approach in many diseases. This study aimed to identify the tumor antigens specifically expressed in tumor cells for lower-grade glioma (LGG) and glioblastoma (GBM) patients.

**Methods:**

In this work, the mRNA microarray expression profile and clinical data were obtained from 301 samples in the Chinese Glioma Genome Atlas (CGGA) database, the mRNA sequencing data and clinical data of 701 samples were downloaded from The Cancer Genome Atlas (TCGA) database. Genetic alterations profiles were extracted from CGGA and cBioPortal datasets. R language and GraphPad Prism software were applied for the statistical analysis and graph work.

**Results:**

PTBP1 and SLC39A1, which were overexpressed and indicated poor prognosis in LGG patients, were selected as tumor-specific antigens for LGG patients. Meanwhile, MMP9 and SLC16A3, the negative prognostic factors overexpressed in GBM, were identified as tumor-specific antigens for GBM patients. Besides, three immune subtypes (LGG1-LGG3) and eight WGCNA modules were identified in LGG patients. Meanwhile, two immune subtypes (GBM1–GBM2) and 10 WGCNA modules were selected in GBM. The immune characteristics and potential functions between different subtypes were diversity. LGG2 and GBM1 immune subtype were associated with longer overall survival than other subtypes.

**Conclusion:**

In this study, PTBP1 and SLC39A1 are promising antigens for mRNA vaccines development in LGG, and MMP9 and SLC16A3 were potential antigens in GBM. Our analyses indicated that mRNA vaccine immunotherapy was more suitable for LGG2 and GBM1 subtypes. This study was helpful for the development of glioma immunotherapies.

**Supplementary Information:**

The online version contains supplementary material available at 10.1186/s41016-022-00301-4.

## Background

Glioma is the most prevalent malignant brain tumor in adult, accounting for 45 ~ 60% of all cases [1]. According to the biological behavior, glioma can be classified into lower-grade glioma (grade II and grade III) and glioblastoma by World Health Organization. The molecular features, including IDH, MGMT, 1p/19q, and so on, were brought into the updated CNS classification in 2016 [2]. Even though numerous studies facilitated the development of glioma, the median overall survival of glioblastoma is 14.6 months, with standard of care surgery, radiotherapy, and chemotherapy [3]. New approaches are needed for glioma patients urgently.

Recently, immunotherapy has revolutionized the treatment of multiple tumors and has been successful for treatment of lung cancer [4], bladder cancer [5], and skin cancer [6]. Tumor-specific antigens (TSAs), silenced in most somatic tissues, are specifically expressed in cancer cells and often evoke strong immune responses [7]. The majority of TSAs were neoantigens that resulted from several genomic aberrations in tumors, such as somatic mutation, copy number aberration, and gene overexpressed [8]. This “tumor-specific” expression pattern in TSA displayed crucial significance for targets in cancer management. Therapeutic mRNA vaccination approaches against human had some encouraging clinical data in influenza virus, Zika virus, rabies virus, and MERS-CoV and SARS‑CoV-2 viruses [9–11]. mRNA vaccine technology provides a new promising era for vaccine technology and had several advantages over DNA vaccines as they induce immune response at a very low concentration, possess high efficacy and safety [12]. Therefore, mRNA vaccines are pivotal target for cancer immunotherapy.

The goal of this study was to dig out the potential mRNA vaccines for LGG and GBM patients, respectively. Two over-expressed, amplified, and mutated genes were discovered as TSAs in LGG (PTBP1 and SLC39A1), and two TSAs were identified in GBM (MMP9 and SLC16A3). The four TSAs were relevant to poor prognosis and antigen presenting cell (APC) infiltration. LGG and GBM samples were clustered based on immune characteristics, and different immune subtypes corresponded to distinct clinical, molecular, and cellular characteristics. Our results indicated that the immune pattern in LGG was not exactly the same as GBM patients. The four candidates might provide a theoretical basis for the development of mRNA vaccines.

## Methods

### Data collection

We obtained the microarray data of 301 samples with clinical and molecular information from CGGA database (http://www.cgga.org.cn) [13]. All patients provided the informed consent and tissue samples were collected during the operation. Two experienced neuropathologists established the pathological diagnosis and molecular pathological testing according to 2016 WHO classification guidelines. Overall survival was calculated from the data diagnosis until death or the end of follow-up. Meanwhile, another RNAseq data of 701 samples with clinical and molecular information were downloaded from TCGA database (https://tcga-data.nci.nih.gov/tcga/) [14].

### cBioPortal analysis

The cBio Cancer Genomics Portal (cBioPortal, http://www.cbioportal.org) was employed to integrate the raw RNA-seq data from TCGA and compare genetic alterations in LGG, GBM, and normal brain [15]. *P* values < 0.05 were considered statistically significant.

### Cell type enrichment analysis

The tumor purity was evaluated by ESTIMATE method using gene expression data. CIBERSORT method was performed to characterize the relative proportions of 22 types of infiltrating immune cells based on gene expression profiles [16].

The relationship between risk score and cell infiltration fraction in glioma was analyzed by Spearman correlation analysis and graphed by R package ‘ggplot2’. The correlation between risk score and different factors was calculated by Pearson analysis and presented by R package ‘corrplot’.

### Functional enrichment analysis

Functional enrichment analysis was performed by Metascape (http://metascape.org) [17]. ssGSEA function with default parameters in GSVA R package was used to calculate the gene enrichment scores.

### Statistical analysis

The association between continuous variable was calculated by Pearson correlation method. Differences between groups were estimated using unpaired Student’s *t* tests, one-way ANOVA, or chi-square tests. The tumor mutational burden (TMB) was calculated using maftools R package. The Kaplan–Meier survival curve and log-rank test were applied using the survminer R package to estimate the distribution of patient overall survival. The hub genes of immune-related genes were identified using WGCNA R package. All figures and statistical analysis were performed based on R language using R software (version 3.6.2). The two-sided *p* value < 0.05 was considered statistically significant.

## Results

### Identification of potential antigens for LGG and GBM

**Fig. 1:**
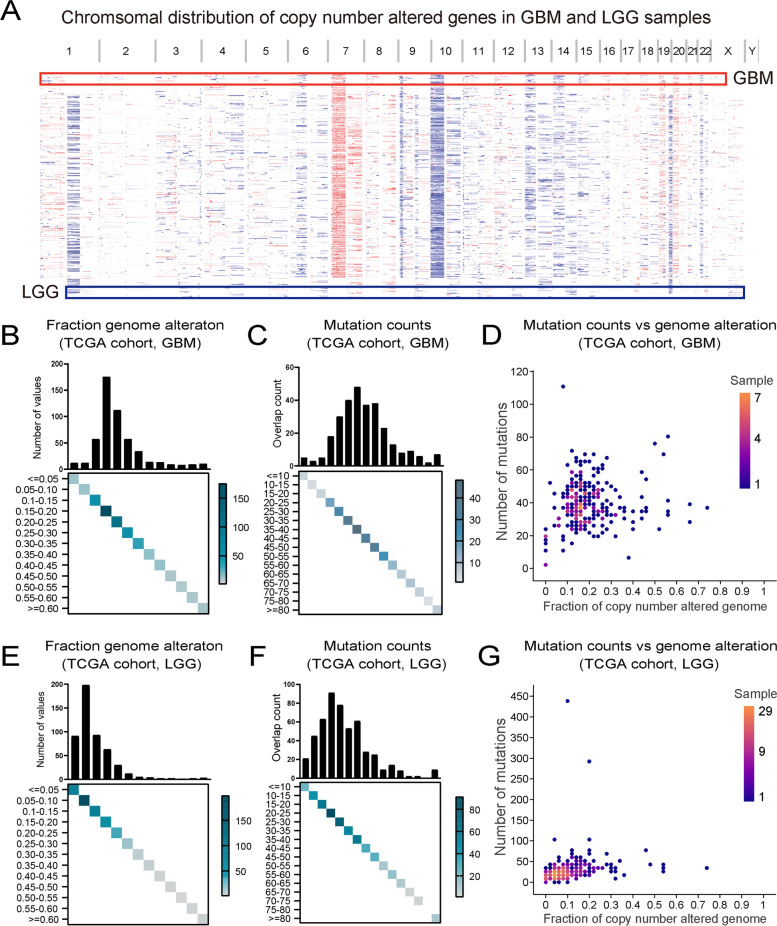
Identification of potential tumor antigens of LGG and GBM. **A** Chromosomal distribution of up- and downregulated genes in LGG and GBM. **B**–**D** The GBM Samples overlapping in altered genome fraction and mutation count groups. **E**–**G** The LGG samples overlapping in altered genome fraction and mutation count groups

Tumor antigens are protein or other intracellular molecular expressed on the surface of tumor cells. In order to identified the specified tumor antigens for LGG and GBM, firstly, we explored differentially expressed genes (DEGs) across LGG/GBM and normal brain in CGGA and TCGA datasets (logFC > 1, *P* < 0.05), respectively. There were 3030 upregulated genes in TCGA LGG patients, 773 upregulated genes in CGGA LGG patients, 4839 upregulated genes in TCGA GBM patients and 1784 upregulated genes in CGGA GBM patients. Subsequently, the copy number aberrated and mutated genes that might encode tumor antigens in LGG and GBM were screened to identify potential tumor antigens (Fig. 1A). GBM patients in TCGA harbored 6817 mutated genes and 13,071 amplified genes, and those patients in CGGA harbored 9208 mutated genes and 7857 amplified genes (Fig. 1B–D). Meanwhile, there were 6693 mutated genes and 15,539 amplified genes in TCGA LGG patients, and 6447 mutated genes and 6763 amplified genes in CGGA LGG patients (Fig. 1E–G). In total, as shown in Table S1, 126 genes in TCGA LGG, 66 genes in CGGA LGG, 458 genes in TCGA GBM and 235 genes in CGGA GBM, which were intersections among three gene lists (over-expressed, mutated, amplified), were found.

### Identification of tumor antigens associated with the clinical outcome of LGG and GBM

**Fig. 2:**
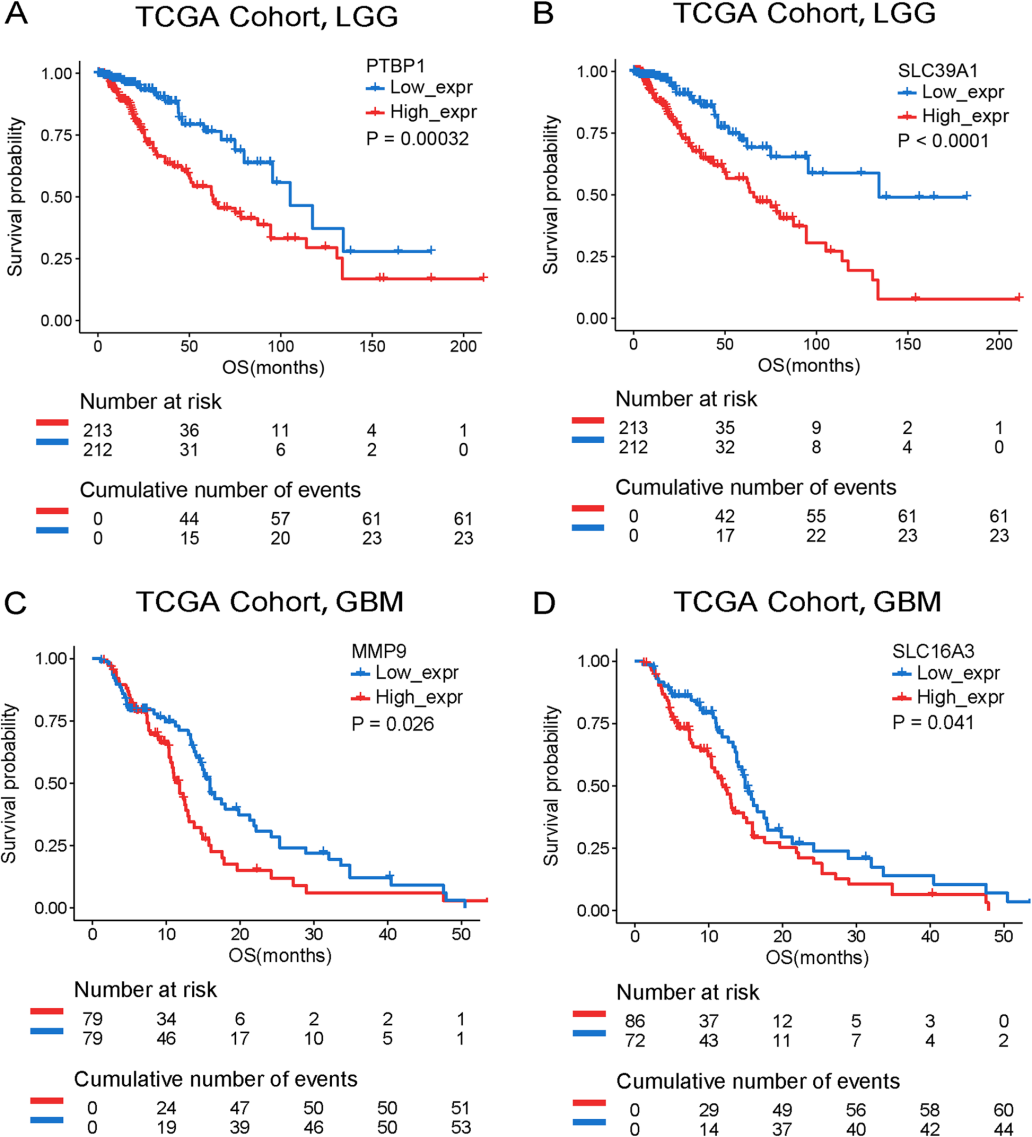
Identification of tumor antigens associated with the clinical outcome of LGG and GBM in TCGA cohort. **A**–**D** Kaplan–Meier curves showing OS of LGG and GBM patients stratified on the basis of **A** PTBP1, **B** SLC39A1, **C** MMP9, and **D** SLC16A3 expression levels

Then, the survival analysis was executed to screen prognosis-related tumor antigens. Two shared genes in LGG (PTBP1 and SLC39A1) and two shared genes in GBM (MMP9 and SLC16A3) were strongly correlated with OS in TCGA (Fig. 2) and CGGA cohorts (Figure S1). LGG patients with higher expression of PTBP1 and SLC39A1 were significantly associated with shorter overall survival, and GBM patients with lower expression of MMP9 and SLC16A3 had a longer OS. Meanwhile, the glioma patients with higher PTBP1, SLC39A1, MMP9, and SLC16A3 expression had shorter OS (*p* < 0.01) (Figure S2 and S3). The results indicated that tumor antigens were crucial with the malignant tumor biological behavior and four genes could serve as potential immunologic and clinical target in LGG and GBM.

### The association between identified tumor antigens and antigen presenting cells

**Fig. 3:**
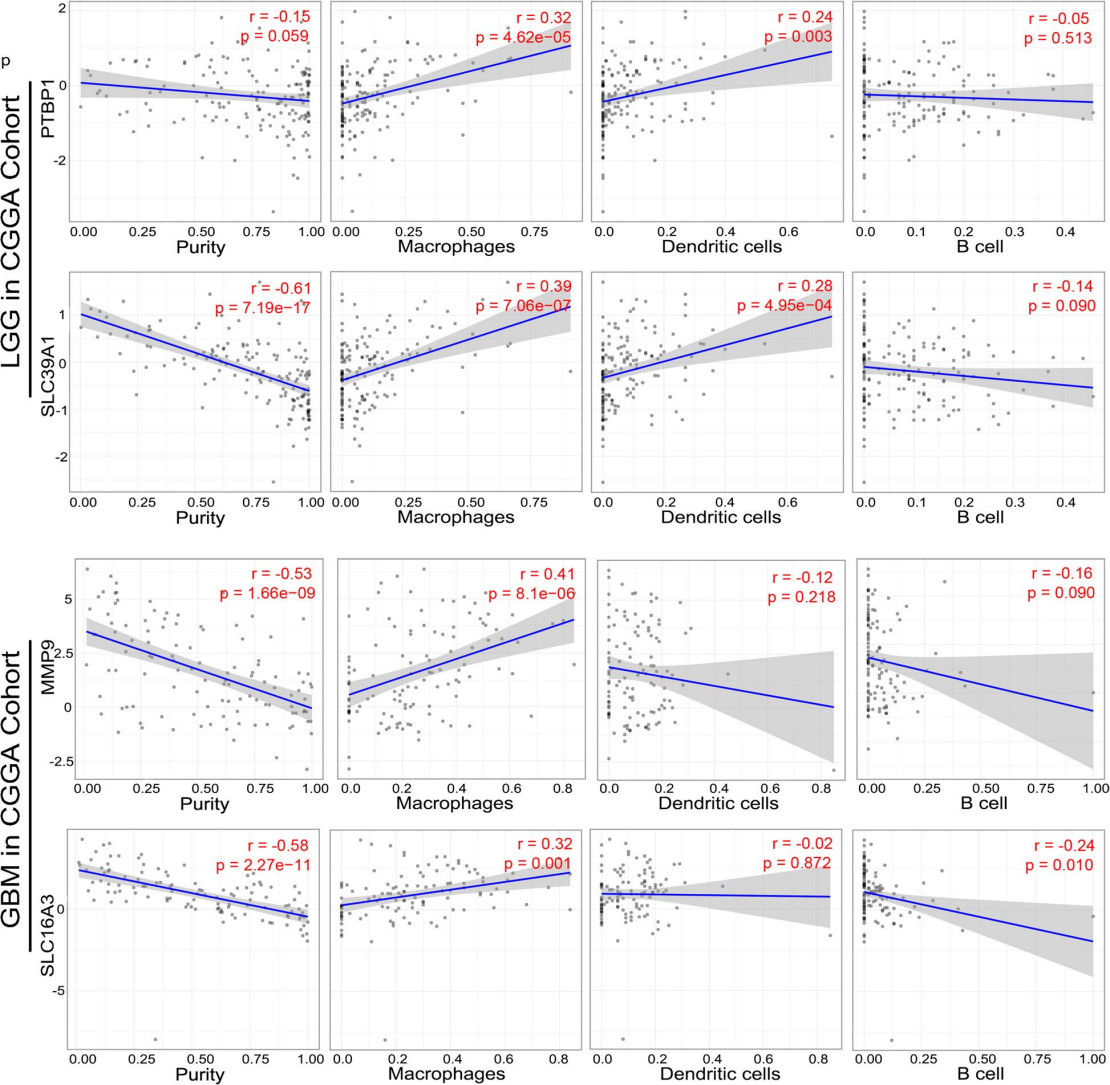
Identification of tumor antigens associated with APCs in CGGA cohort. Correlation between the expression levels of **A** PTBP1, **B** SLC39A1, **C** MMP9, and **D** SLC16A3 and infiltration of macrophages, dendritic cells and B cells in LGG and GBM

Professional antigen presenting cells, including macrophages, B cells, and dendritic cells, process antigen bound on its cell surface and present antigens to T cells [18]. The correlation analysis was applied to reveal the relationship between APCs and tumor antigens. As shown in Fig. 3, in LGG, the mRNA expression of PTBP1 and SLC39A1 was found positively related to the abundance of macrophages (PTBP1, *r* = 0.32; SLC39A1: *r* = 0.39), and DCs (PTBP1: *r* = 0.24; SLC39A1: *r* = 0.28; *P* < 0.05). Meanwhile, the mRNA expression of PTBP1 and SLC39A1 was significantly negative related to tumor purity (PTBP1, *r* =  − 0.15; SLC39A1: *r* =  − 0.61; *P* < 0.05). In GBM, the mRNA expression of MMP9 and SLC16A3 was only positively related to the abundance of macrophages (PTBP1, *r* = 0.41; SLC16A3: *r* = 0.32; *P* < 0.05). And the mRNA expression of MMP9 and SLC16A3 was significantly negative related to tumor purity (PTBP1, *r* =  − 0.53; SLC39A1: *r* =  − 0.58; *P* < 0.05). Similar results were found in TCGA dataset (Figure S4). These discoveries suggested that macrophages performed a dominate role in recognizing and presenting tumor antigens in LGG or GBM. Moreover, the DCs also served as sensors in activating innate immune response in LGG. Taken together, the tumor antigens identified for LGG and GBM vaccine exhibited potential potency in antigen presentation and inducing potent tumor specific immunity.

### Development immune subtypes of LGG and GBM

**Fig. 4:**
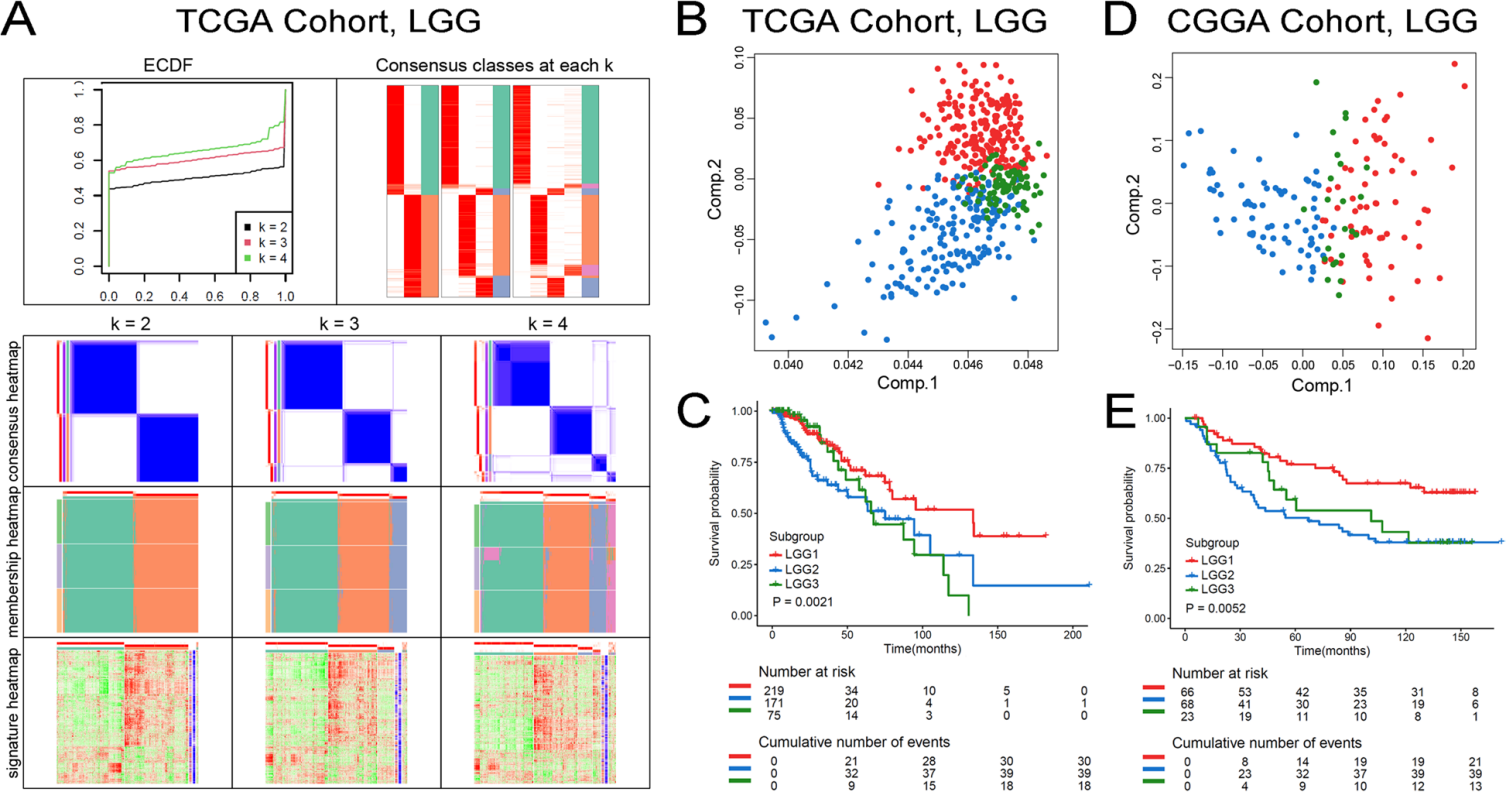
Consensus clustering of LGG samples. **A** Color-coded heatmaps corresponding to the consensus matrices for *k* = 2 to *k* = 4. **B** PCA of three groups (*k* = 3) based on gene expression data in TCGA. **C** Kaplan–Meier curves survival based on the three LGG immune subtypes in the TCGA cohort. **D** PCA of three groups (*k* = 3) based on gene expression data in CGGA. **E** Kaplan–Meier curves survival based on the three LGG immune subtypes in the CGGA cohort

**Fig. 5:**
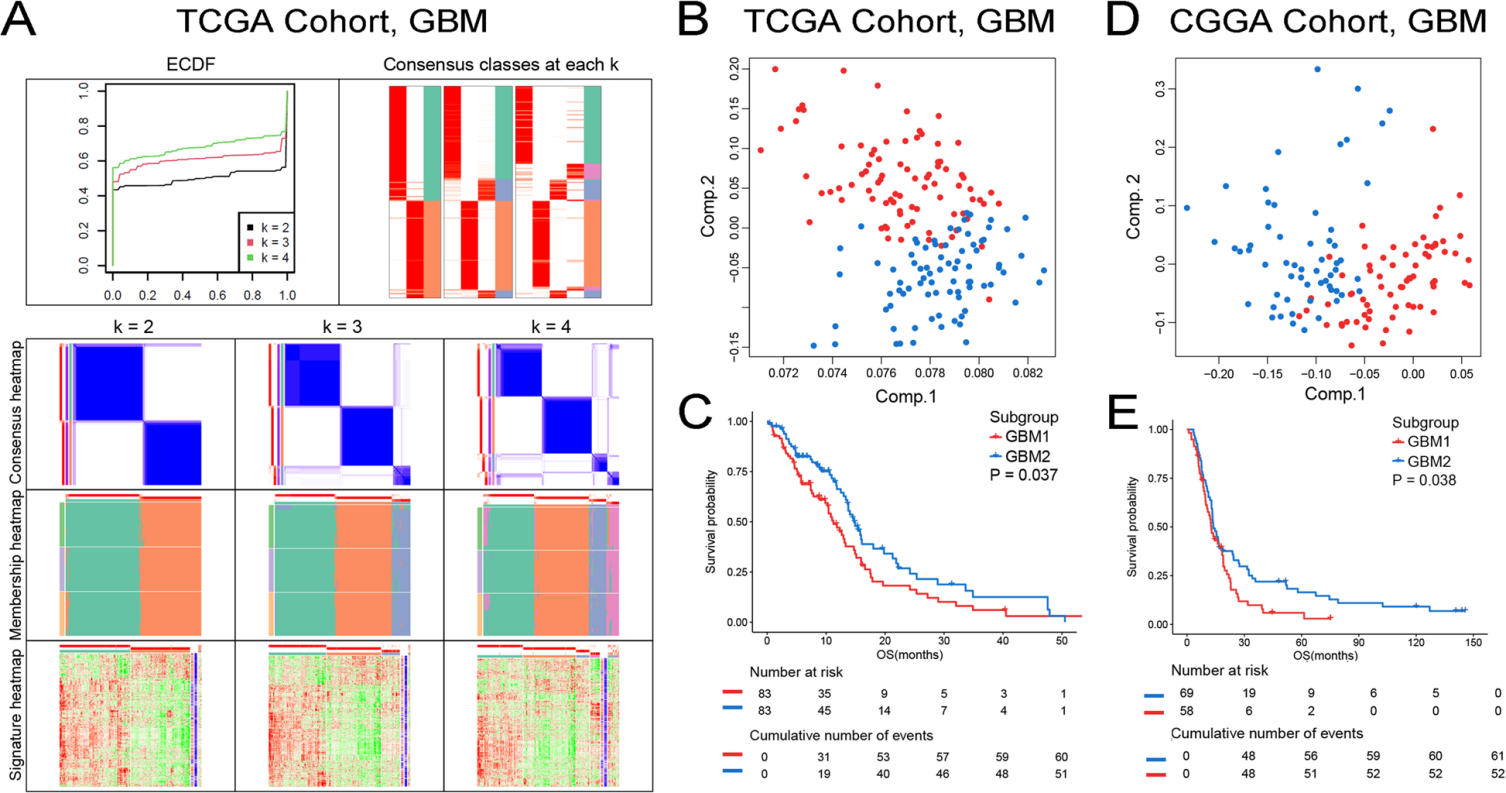
Consensus clustering of GBM samples. **A** Color-coded heatmaps corresponding to the consensus matrices for *k* = 2 to *k* = 4. **B** PCA of two groups (*k* = 2) based on gene expression data in TCGA. **C** Kaplan–Meier curves survival based on the two GBM immune subtypes in the TCGA cohort. **D** PCA of two groups (*k* = 2) based on gene expression data in CGGA. **E** Kaplan–Meier curves survival based on the two GBM immune subtypes in the CGGA cohort

To characterize the immune state of LGG and GBM, we enrolled 1997 immune-related genes and applied cluster analysis to identify discrete groups in TCGA and CGGA datasets. In the spherical k-means clustering analysis, the ECFD curves, consensus classes, and consensus heatmap were used to identify the optimal cluster number in TCGA LGG (Fig. 4A) and GBM (Fig. 5A) cohorts, respectively. The results indicated that *k* = 3 was more stable for LGG immune-related clustering (LGG1-LGG3) (Fig. 4A). And the PCA analysis, conducting to estimate the immune subtypes, supported the three immune modules classification scheme (Fig. 4B, D). Similar results were found in the CGGA cohort (Figure S5). LGG patients in LGG2 and LGG3 subtypes were more likely to have a worse prognosis, and patients in LGG1 group were associated with best survival probability in TCGA and CGGA (Fig. 4C, E). Meanwhile, we uncovered that the classification of GBM patients into two immune subtypes (GBM1-GBM2) was more suitable than other classification scheme (Fig. 5A). The PCA analysis revealed that the samples in GBM1 and GBM2 dispersed in different segments clearly (Fig. 5B, D). The survival analysis found that cases in GBM1 had a significant shorter OS than patients in GBM2 subtype in TCGA and CGGA (Fig. 5C, E). Therefore, the immune module could promote the prognosis prediction for LGG and GBM.

### Cellular and molecular characteristics of the immune subtypes in LGG and GBM

**Fig. 6:**
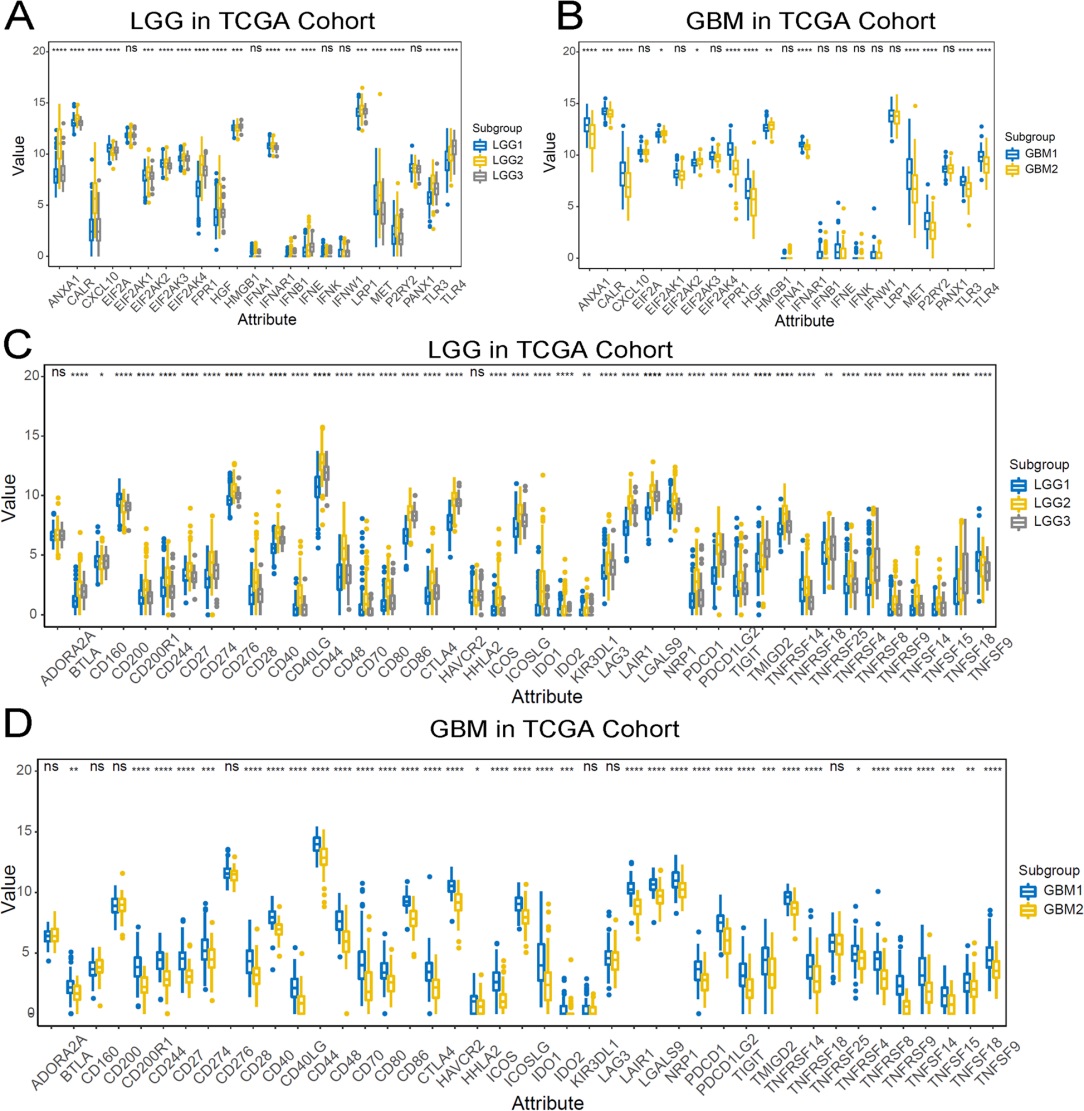
Association between immune subtypes and ICPs and ICD modulators. **A**, **C** Differential expression of ICP genes and ICD genes among the LGG immune subtypes in TCGA cohorts. **B**, **D** Differential expression of ICP genes and ICD genes among the GBM immune subtypes in TCGA cohorts. *p < 0.01, **p < 0.001, ***p < 0.0001, and ****p < 0.00001

To gain the insight of immune heterogeneity in different immune subtypes of LGG and GBM, the expression level of 45 immune checkpoints (ICPs) and 25 immunogenic cell death (ICD) regulators were detected in TCGA cohort. In LGG patients, the expression of ANXA1, CALR, CXCL10, EIF2A, EIFF2AK3, EIFF2AK4, FPR1, HGF, HMGB1, IFNAR1, IFNB1, IFNE, LRP1, MET, P2RY2, and TLR3 were significantly upregulated in LGG2 immune subtype, while TLR4 was significantly upregulated in LGG3 subtype (Fig. 6A). Furthermore, similar to protumor immune cells, most immune checkpoints were highly expressed in LGG2 immune subtypes. In addition, CD200 and TNFRSF9 were significantly upregulated in LGG1 (Fig. 6C). In GBM patients, the expression of ANXA1, CALR, CXCL10, FPR1, HGF, IFNAR1, MET, P2RY2, TLR3, and TLR4 were significantly upregulated in GBM1 immune subtype, while EIFF2AK3 was significantly upregulated in GBM2 (Fig. 6B). Besides, the expression level of 38 ICPs, except ADORA2A, CD160, CD200, CD276, KIR3DL1, LAG3, and TNFRSF25 were significantly upregulated in GBM1 (Fig. 6C). Some researches have shown that TMB may affect the patients’ response to immunotherapy [19]. LGG patients with subtype LGG2 were found to have a significantly higher TMB than LGG1 and LGG3 subtypes (*P* < 0.05, Figure S6A). However, the TMB was not significantly different between the two immune subtypes in GBM patients (Figure S6B).

**Fig. 7:**
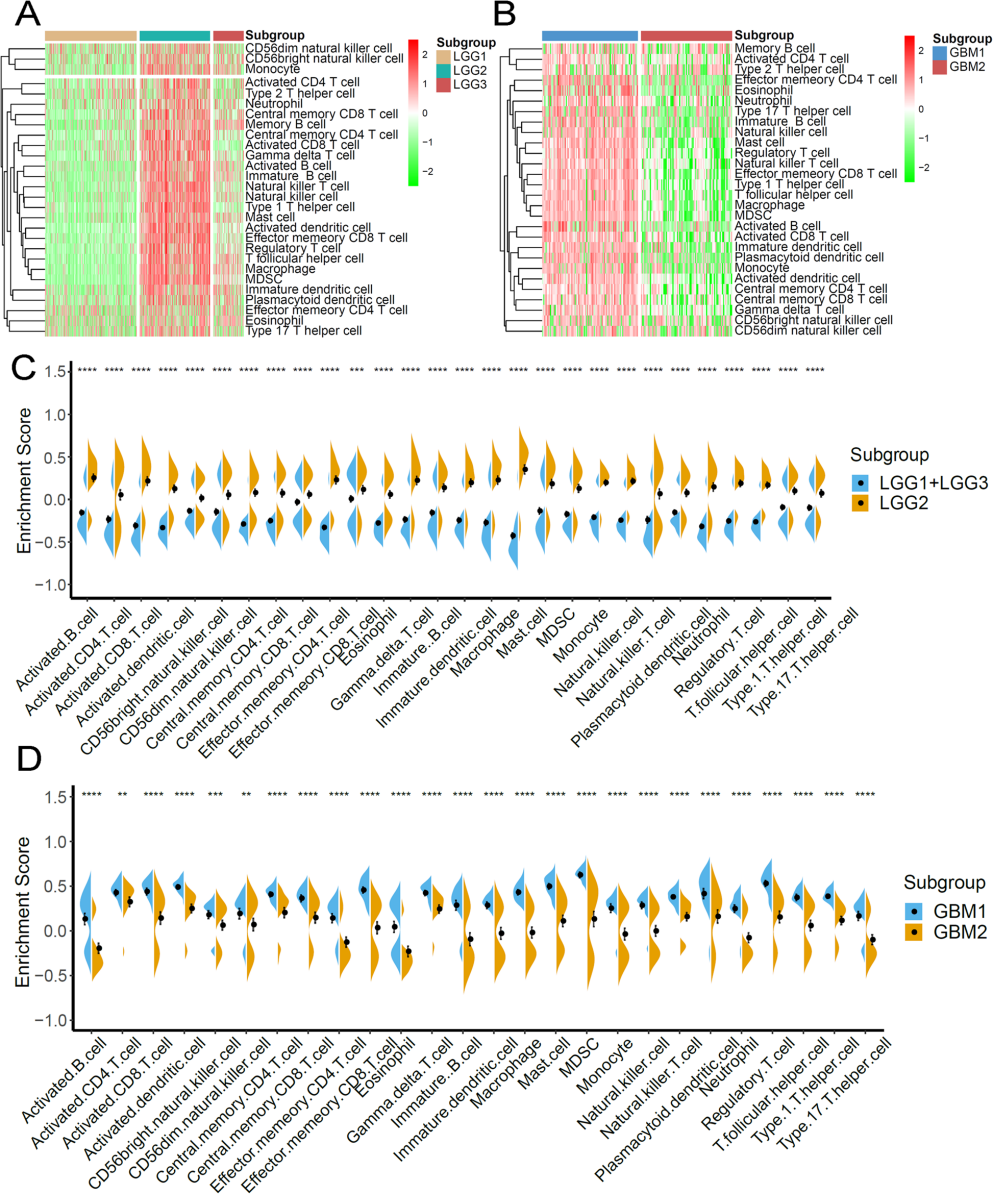
Cellular and molecular characteristics of immune subtypes. **A**, **B** Differential enrichment scores of 28 immune cell signatures among LGG and GBM immune subtypes. **C**, **D** Differential enrichment scores of 28 immune cell signatures in LGG and GBM

Several recently published studies elicited the clinical value of mRNA vaccines in treating cancer depended on innate and adaptive immune states [20]. The ssGSEA method was adopted to calculate the score of 28 immune cells in LGG and GBM samples to identify the characteristics the association between immune component and immune subtypes. As shown in Fig. 7A, the infiltration of immune cells in microenvironment indicated the significant differences between immune subtypes in LGG. Of note, LGG1 and LGG3 had lower score of immune cells, suggesting an immune-cold subtype, while LGG2 exhibited higher immune cell score, suggesting an immune-active subtype. The enrichment score of immune cells, especially the protumor immune cells (CD56dim NK cells, immature dendritic cells, myeloid-derived suppressor cells, neutrophils, plasmacytoid DCs, regulatory T cells, and macrophages) were enriched in subtype LGG2, compared to the other subtypes (*P* < 0.0001, Fig. 7C), suggesting that subtype LGG2 was characterized by the protumor microenvironment in LGG. Meanwhile, we detected the distinct immune states between two GBM immune subtypes. The GBM1 had significant higher enrichment scores for all immune cells. The results suggested that the mRNA vaccine therapy was likely to evoke the immune response for patients in LGG2 and GBM1 subtype.

### Identification of immune network of LGG and GBM

**Fig. 8:**
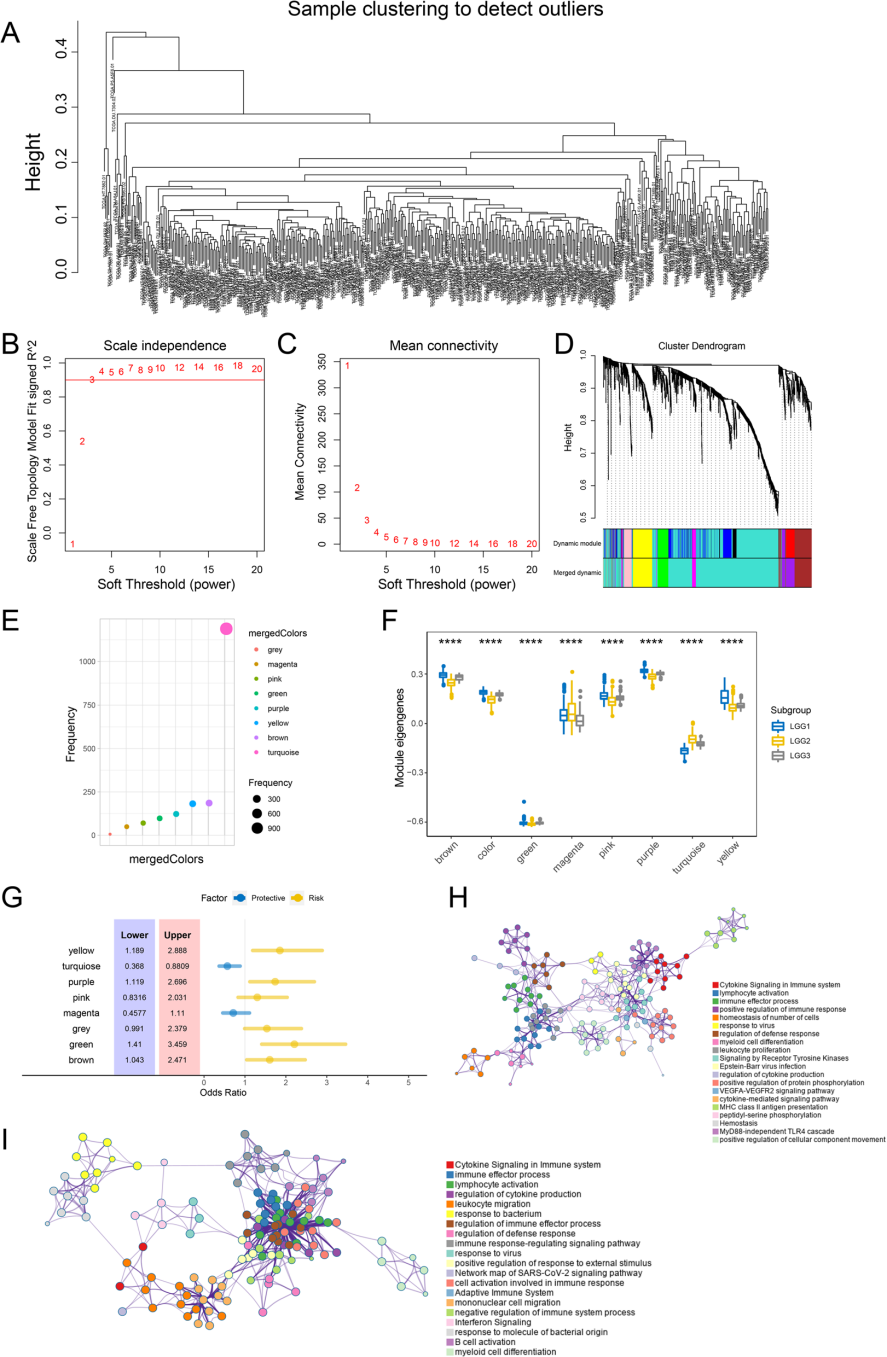
Identification of immune gene co-expression modules of LGG. **A** Determination of the scale-free fit index. **B** Scale-free fit index for various soft-thresholding powers (β). **C** Mean connectivity for various soft-thresholding powers. **D** Treemap of all immune-related genes clustered based on the TOM matrix. **E** Gene numbers in each module. **F** Differential distribution of module eigengenes in LGG subtypes. **G** Forest maps of single factor survival analysis of 8 modules of LGG. **H** GO analysis for hub genes from green, purple, and yellow modules. **I** GO analysis for hub genes from turquoise modules

To reveal the gene co-expressed module in LGG and GBM, we performed WGCNA analysis. In LGG samples, as shown in Fig. 8A–C, we selected β = 3 as the soft thresholding power to build a scale-free network. The gene dendrogram was generated by mean linkage hierarchical clustering. The colored rows on the bottom of the tree diagram showed the module assignments determined by dynamic tree cutting. The eigengenes of every module were computed by merging the closed modules into new modules with height = 0.25, deep split = 2, and minimum module size = 30. According to the gene co-expressed gene pattern, eight gene modules were identified, including brown, green, grey, magenta, pink, purple, turquoise, and yellow modules (Fig. 8D, E). The eigengenes of magenta and turquoise modules were significantly higher in LGG2 (Fig. 8F). In addition, the clinical analysis of gene co-expressed network showed that the green, purple, and yellow modules were significantly correlated with unfavorable prognosis in LGG patients, while the turquoise module was associated with longer overall survival (Fig. 8G). Moreover, the gene ontology analysis showed that hub genes in green, purple, and yellow modules were all enriched in ‘immune system response’, ‘immune effector process,’ ‘innate immune response,’ and ‘adaptive immune response’ (Fig. 8H). However, the hub genes in turquoise modules were significantly associated with “pathways to cancer,” “regulation of I-kappaB kinase/NF-kappaB signaling,” and “signaling by receptor tyrosine kinases” (Fig. 8I).

**Fig. 9:**
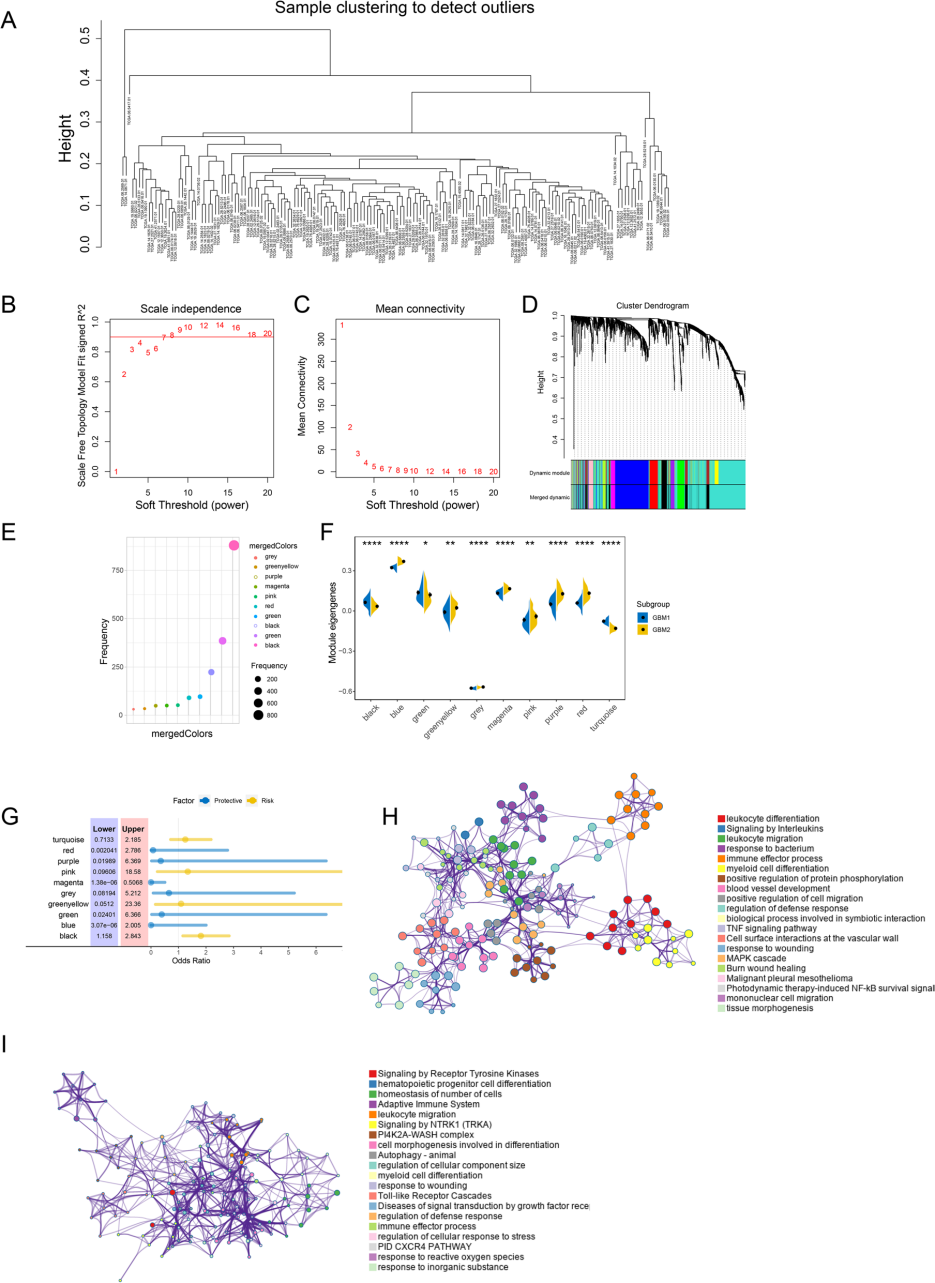
Identification of immune gene co-expression modules of GBM. **A** Determination of the scale-free fit index. **B** Scale-free fit index for various soft-thresholding powers (β). **C** Mean connectivity for various soft-thresholding powers **D** Treemap of all immune-related genes clustered based on the TOM matrix. **E** Gene numbers in each module. **F** Differential distribution of module eigengenes in LGG subtypes. **G** Forest maps of single factor survival analysis of 10 modules of GBM. **H** GO analysis for hub genes from magenta modules. **I** GO analysis for hub genes from black modules

Meanwhile, the β = 7 was selected as the soft thresholding power in constructing the network for GBM patients (Fig. 9A–C). GBM1 samples were significantly correlated to black, green and turquoise modules, while GBM2 samples were associated with blue, greenyellow, grey, magenta, pink, purple, and red (Fig. 9D–F). The forest plot exhibited that magenta module was a protective factor for GBM patients and black module was a risk factor for GBM patients (Fig. 9G). According to the GO biological process enrichment analysis, we found that the genes in magenta module were highly enriched in “signaling by receptor tyrosine kinases,” “hematopoietic progenitor cell differentiation,” “signaling by NTRK1 (TRKA),” and so on (Fig. 9H). The hub genes in black module were mainly related to “leukocyte differentiation,” “signaling by interleukins,” “cytokine signaling in immune system leukocyte migration,” “response to bacterium,” and so on (Fig. 9I). These results indicated that the application pattern for mRNA vaccines may be helpful for the treatment for glioma in the future.

## Discussion

Glioma are the most prevalent brain malignant tumor in adults [21]. Current treatment for glioma is mainly donated by surgical resection followed by radiotherapy and chemotherapy. However, due to the toxicity to normal cell, resistance to temozolomide and high rate to recurrent, the treatment is limited [22, 23]. mRNA vaccine, which could activate innate immune activation-mediated co-stimulation, is one important approach in immunotherapy [24]. One of the challenge and bottleneck for manufacturing mRNA vaccine for glioma was to select antigens uniquely overexpressed in cancer cells.

Here, we combined the gene expressed pattern, mutation profiles, and copy number aberration to detect TSA genes as immunological and clinical targets in glioma. Tumor antigens are mainly derived from upregulated and mutated genes. In CGGA and TCGA datasets, the tumor-normal comparisons were executed to find out the over-expressed, amplified, and mutated genes in LGG and GBM, respectively. Then the Kaplan–Meier analysis was conducted to explain the clinical significance of candidate tumor-specific antigens. We found the expression of PTBP1 and SLC39A1 in LGG and MMP9 and SLC16A3 in GBM were significantly associated with worse OS in CGGA and TCGA cohorts. The results indicated that the development of mRNA vaccines targeted these four genes may prolong the survival time of glioma patients.

Antigen-presenting cells (APCs), a heterogeneous group of immune cells, mediated the cellular immune response by processing and presenting antigens for T cells, including macrophages, B cells and dendritic cells. The correlation analysis of APCs and TSA genes indicated that the expression of PTBP1 and SLC39A1 were significantly positively associated with high APC infiltration, especially macrophages and DCs. Meanwhile, the expression of MMP9 and SLC16A3 were significantly positively associated with macrophages infiltration. The macrophages, composed of bone marrow-derived macrophages (BMDMs) and brain-resident microglia (MG), constituted the most abundant immune cell population in the tumor microenvironment in glioma [25]. The macrophages have been revealed as key role for the progression of glioma. Our results indicated that the amount and subtype of macrophage had critical effect on antigen presenting in glioma, especially GBM.

These results indicated that PTBP1, SLC39A1, MMP9, and SLC16A3 potentially played vital roles in immunity as TSAs. Numerous studies have shown that PTBP1, a protein coding gene, played essential roles in various cancers, including colorectal cancer, renal cell cancer, breast cancer, and glioma [26]. The PTBP1 performed functions through regulating of glycolysis, apoptosis, proliferation, tumorigenesis, invasion, and migration [27]. SLC39A proteins are ZIP metal ion transport proteins which mainly expressed on the plasma membrane in various tissues [28]. SLC39A1 may play an important role in tumor progression [29]. SLC39A1 could significantly decreased the level of Zn2 + in cancer tissue, thereby reducing the level of citrate, and ultimately resulting in the malignant progression of prostate cancer and glioma [30]. MMP-9, a member of the matrix metalloproteinases (MMP) family known to confer invasive behavior to cancer cells. MMP9 is highly expressed in cancer and played crucial roles in carcinogenesis and progression [31]. SLC16A3 is a member of the proton-linked monocarboxylate transporter (MCT) family. Previous studies have confirmed that hypoxia can induce SLC16A3 expression through HIF-1 pathway and participate in the occurrence and development of tumors [32, 33]. These results clearly suggested that they may be potential targets for mRNA vaccines.

mRNA vaccines are a type of treatment that active the immune system to fight cancer cells and induced long-term remission only in the minority of patients. We categorized LGG and GBM into different immune subtypes based on immune gene expression profiles for selecting the population suitable for vaccination. Each immune subtype corresponded to distinct clinical, molecular and cellular characteristics. In LGG patients, LGG2 was shown to have a better prognosis. The expression level of ICD modulators and ICPs were significantly upregulated in LGG2 patients. Meanwhile, LGG2 showed significantly elevated scores in antigen-presenting cells and cytolytic T lymphocytes. In GBM patients, GBM1 was shown to have a better prognosis. The expression level of ICD modulators and ICPs were significantly upregulated in GBM1 patients. Meanwhile, GBM1 showed significantly elevated scores in antigen-presenting cells and cytolytic T lymphocytes. These results indicated that mRNA vaccine could be more effective in patients in LGG2 and GBM1.

Compared to the studies conducted by Quanwei Zhou et al. [34], Shuai Ma et al. [35], and Hua Zhong [36], the approach for selecting antigen genes in this study exhibited more advantages. We used normal brain samples as control group to find out specific antigen genes in LGG and GBM patients in multi-platform. In this study, limited by the retrospective nature of our study, a large-scale prospective study is needed to evaluate the clinical significance of the four potential antigen genes.

## Conclusions

In conclusion, we identified PTBP1and SLC39A1, and MMP9 and SLC16A3 as tumor specific antigens for LGG and GBM, respectively. They may be used as possible targets for mRNA vaccine therapy to help boost the body’s immune system to kill more cancer cells. The patients in LGG2 or GBM1 subtypes were potentially benefit more from mRNA vaccines. Thus, this study provides a theoretical foundation for mRNA vaccine against glioma and defines suitable vaccination patients.

## Supplementary Information

**Additional file 1: Supplementary Figure 1.**

**Additional file 2: Supplementary Figure 2.**

**Additional file 3: Supplementary Figure 3.**

**Additional file 4:** **Supplementary Figure 4.**

**Additional file 5: Supplementary Figure 5.**

**Additional file 6: Supplementary Figure 6.**

**Additional file 7: Supplementary Table S1.** The gene list of potential antigens for LGG and GBM in TCGA and CGGA cohorts.

## Data Availability

The sequencing data, clinical, and follow-up information of LGG and GBM patients were uploaded to the CGGA portal (http://cgga.org.cn/). All datasets used and/or analyzed in this study are available from the corresponding author on reasonable request.
