# Supplementary figures and images for: Identification of tumor-associated antigens and immune subtypes of lower-grade glioma and glioblastoma for mRNA vaccine development
Source: Chin Neurosurg J. 2022 Oct 28;8:34. doi: 10.1186/s41016-022-00301-4 (PMC9614757; doi:10.1186/s41016-022-00301-4)

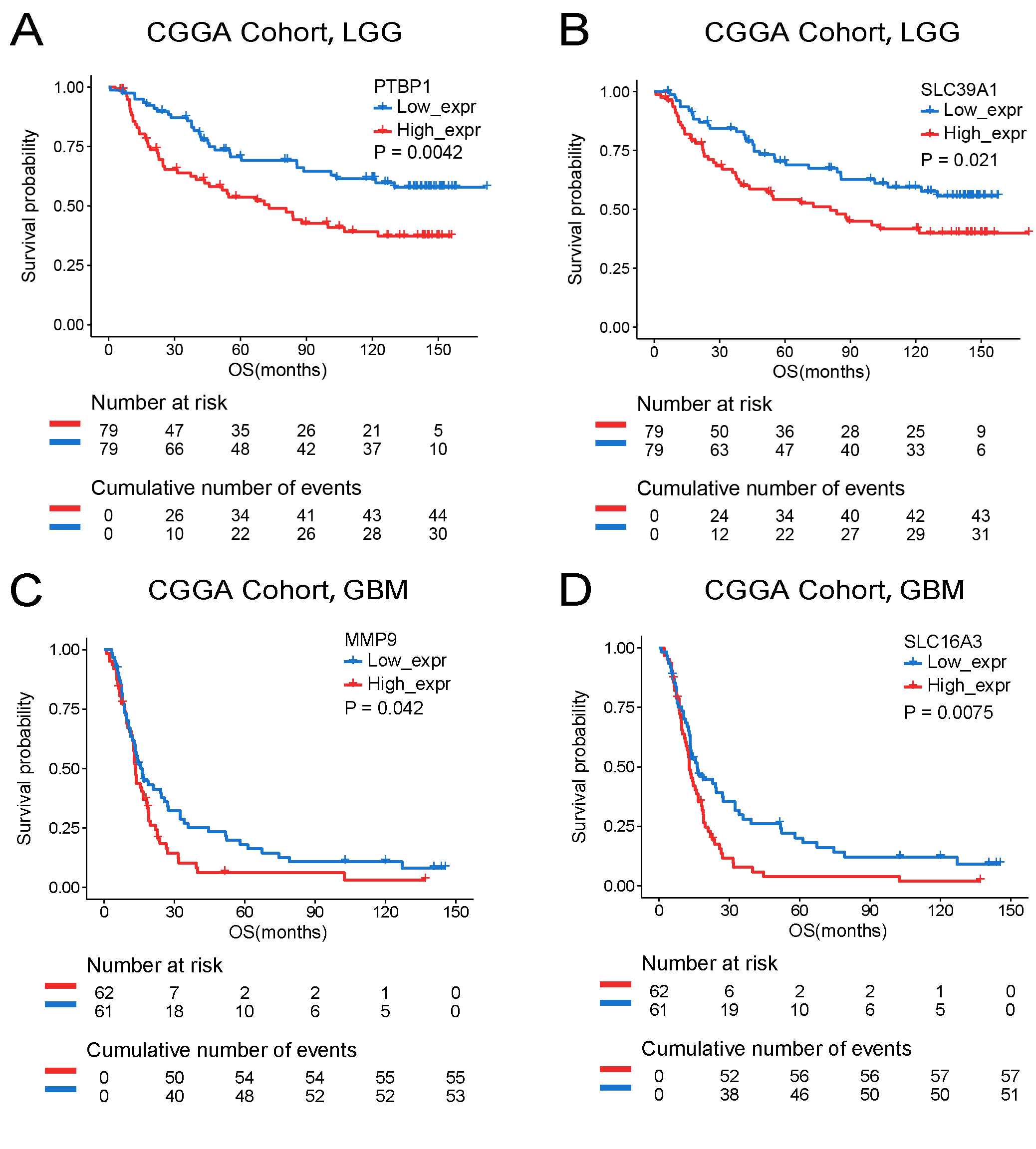

Supplement: Supplementary file 1 — Additional file 1: Supplementary Figure 1. [file 41016_2022_301_MOESM1_ESM.tif]

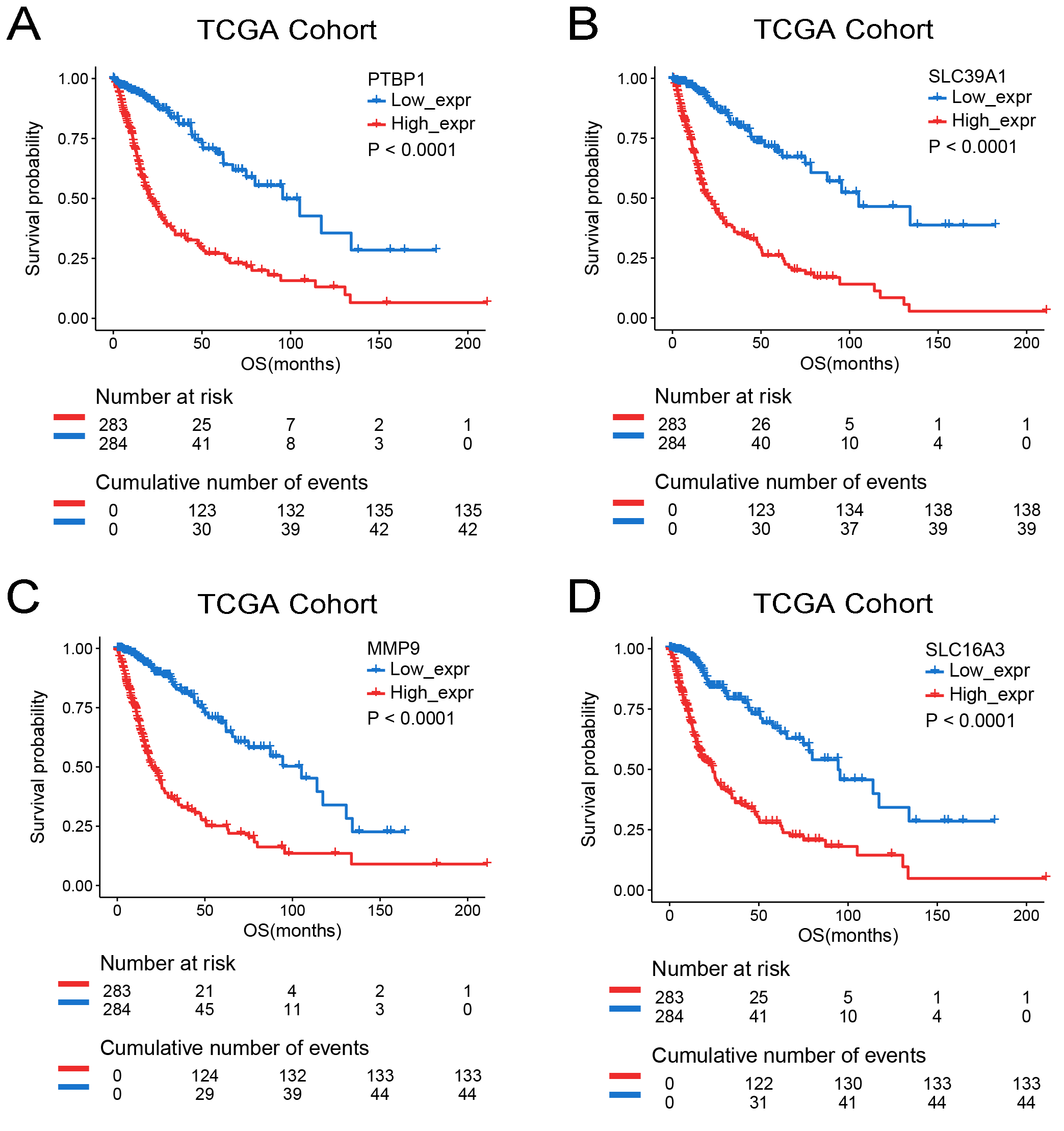

Supplement: Supplementary file 2 — Additional file 2: Supplementary Figure 2. [file 41016_2022_301_MOESM2_ESM.tif]

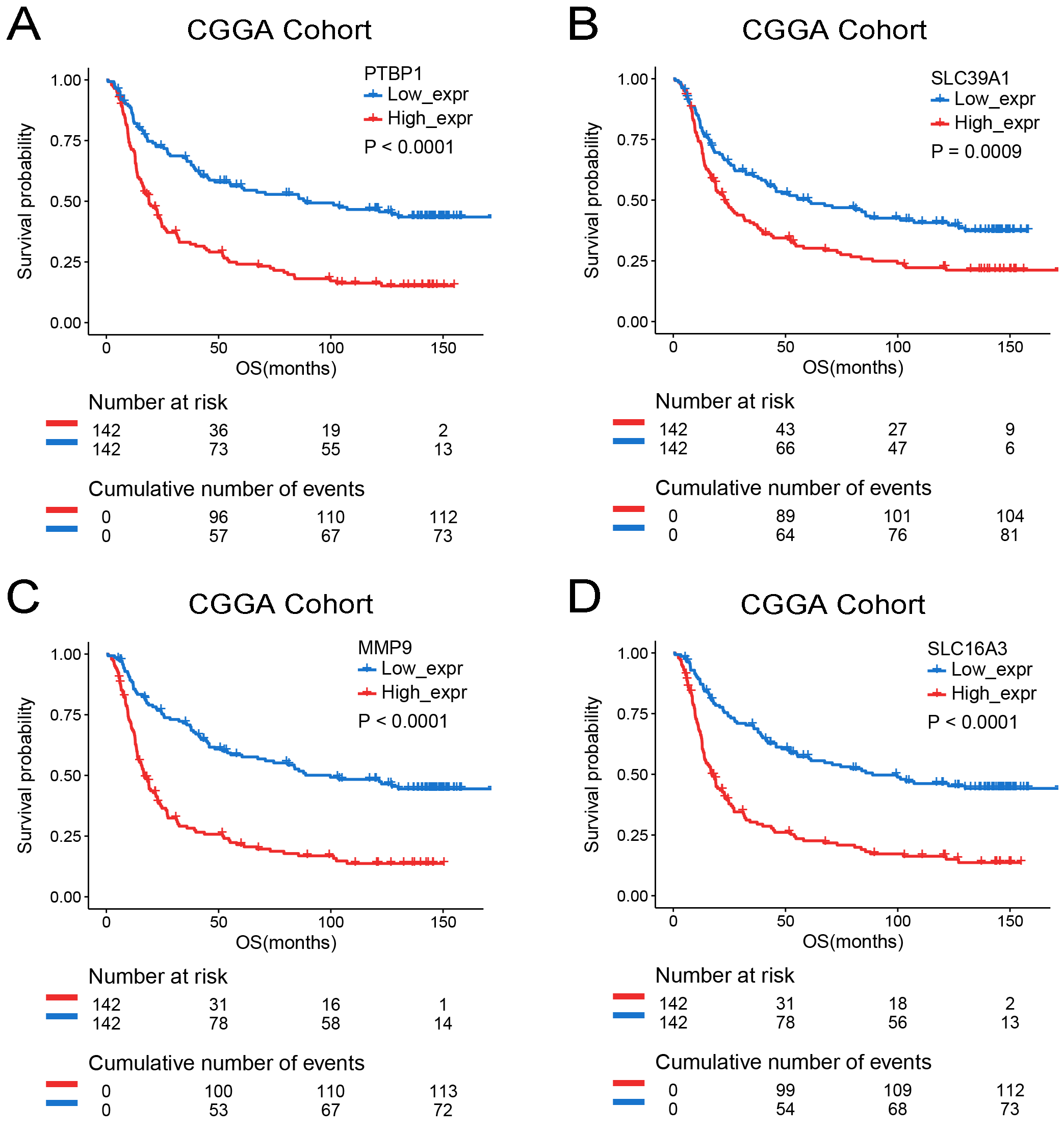

Supplement: Supplementary file 3 — Additional file 3: Supplementary Figure 3. [file 41016_2022_301_MOESM3_ESM.tif]

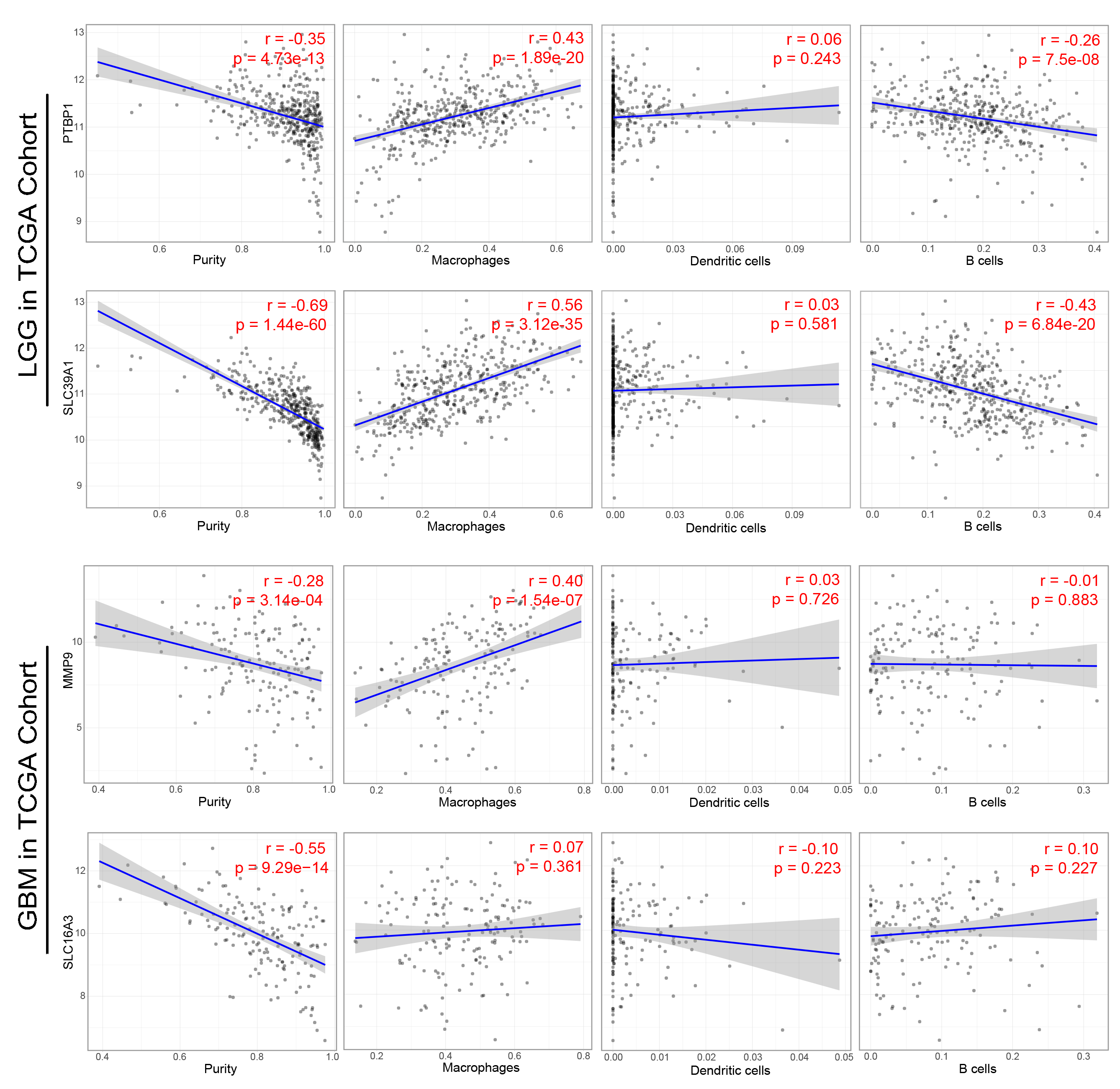

Supplement: Supplementary file 4 — Additional file 4: Supplementary Figure 4. [file 41016_2022_301_MOESM4_ESM.tif]

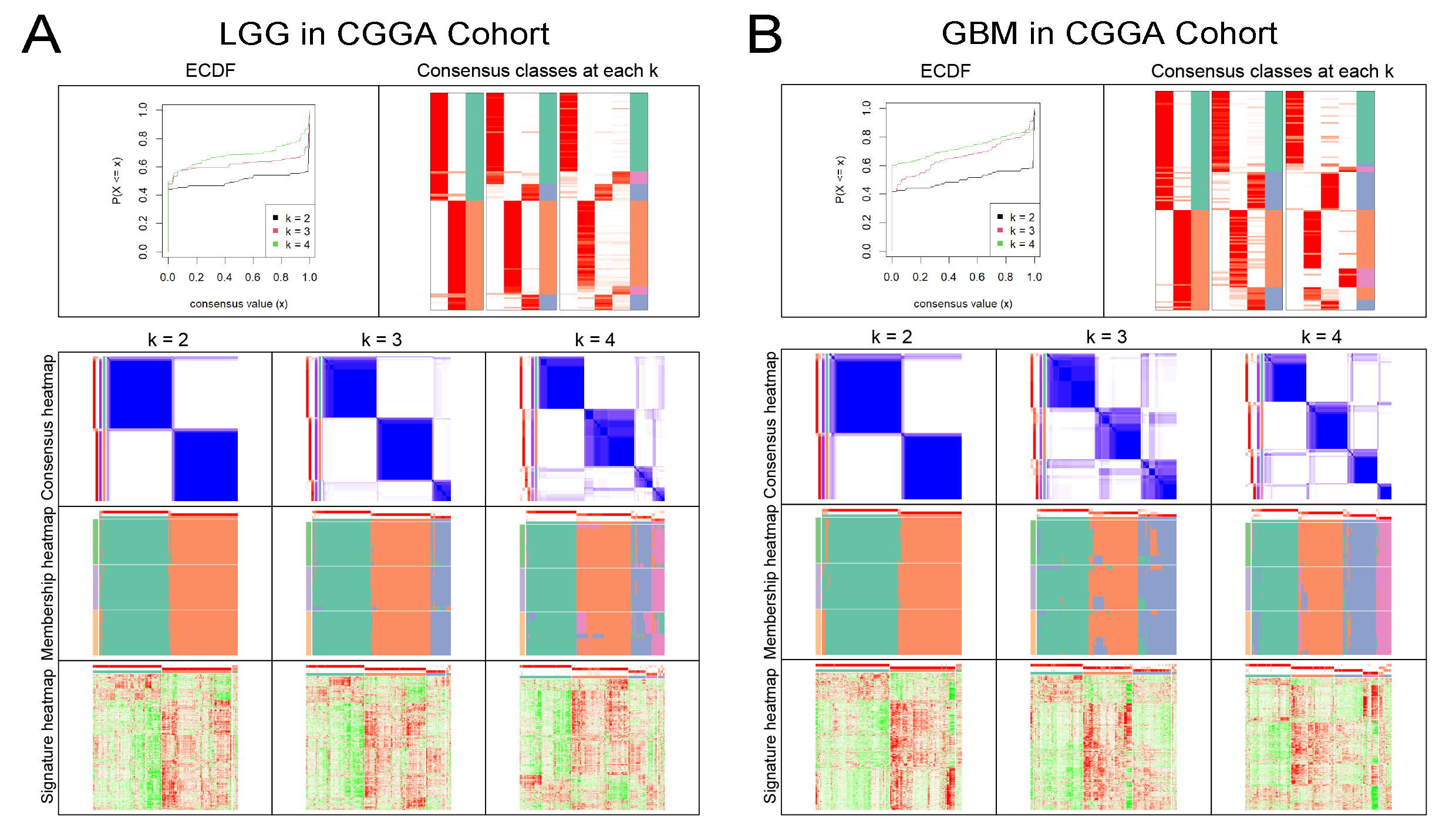

Supplement: Supplementary file 5 — Additional file 5: Supplementary Figure 5. [file 41016_2022_301_MOESM5_ESM.tif]

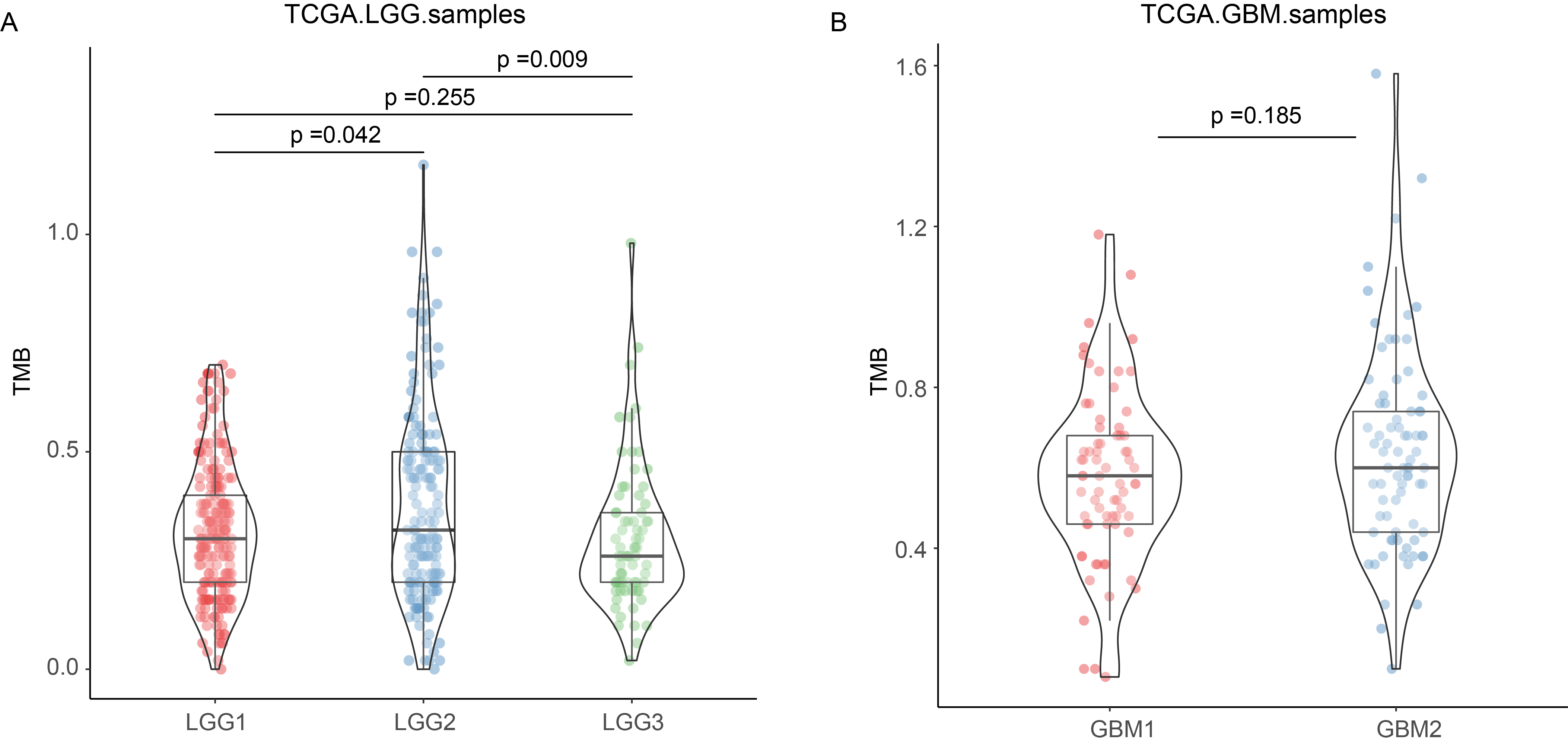

Supplement: Supplementary file 6 — Additional file 6: Supplementary Figure 6. [file 41016_2022_301_MOESM6_ESM.tif]
