# Supplementary material for: Identification of tumor-associated antigens and immune subtypes of lower-grade glioma and glioblastoma for mRNA vaccine development
Source: Chin Neurosurg J. 2022 Oct 28;8:34. doi: 10.1186/s41016-022-00301-4 (PMC9614757; doi:10.1186/s41016-022-00301-4)
Supplement: Supplementary file 7 — Additional file 7: Supplementary Table S1. The gene list of potential antigens for LGG and GBM in TCGA and CGGA cohorts. [file 41016_2022_301_MOESM7_ESM.docx]

Table S1. The gene list of potential antigens for LGG and GBM in TCGA and CGGA cohorts.

| CGGA LGG patients | CGGA GBM patiens | | TCGA LGG patients | | TCGA GBM patiens |
| --- | --- | --- | --- | --- | --- |
| USP49 | HIST1H1B | | KIF4A | | MMP9 |
| ZNF692 | EZH2 | | LAMC1 | | NCAPG |
| UNC5CL | HOXA7 | | ASPM | | BUB1 |
| TRAIP | TACC3 | | KNTC1 | | KIF4A |
| COL11A2 | NES | | KIF2C | | ABCC3 |
| TCF7L1 | IGF2BP3 | | PDIA4 | | CDCA2 |
| UHRF1 | IFI30 | | ERCC6L | | ADAMDEC1 |
| DDR1 | PLEKHG2 | | SKA1 | | SPOCD1 |
| PTPRZ1 | ABCC3 | | PYGL | | COL4A1 |
| C1QTNF6 | COL1A1 | | CDCA7L | | CD163 |
| BEND7 | BUB1 | | CEP55 | | NUF2 |
| CDK4 | SAMD9L | | NCAPG | | HOXA2 |
| CPXM1 | PTBP1 | | MCM3 | | ESPL1 |
| AMH | NCAPH | | SERPINH1 | | EZH2 |
| MST1 | GDF15 | | CENPI | | NOX4 |
| MAP3K8 | TBX2 | | JAG1 | | COL1A1 |
| LAMA5 | SFRP4 | | NUP37 | | HOXC9 |
| REC8 | HLA-DMB | | FANCI | | TNC |
| LCAT | SLC16A3 | | ASF1B | | NCAPH |
| IGLL1 | S100A10 | | KIF20A | | ST14 |
| TGM1 | BTN3A2 | | PLK1 | | NR5A2 |
| RTKN | PILRB | | RAD51AP1 | | KIF15 |
| CPNE2 | CCNE2 | | TACC3 | | MMP7 |
| KIF18B | CATSPER1 | | NUF2 | | RCC1 |
| TNFRSF6B | CD163 | | FANCD2 | | NKX3-2 |
| CD82 | HIST1H1D | | DLGAP5 | | TMSB15A |
| HAUS8 | CD93 | | CHEK2 | | LEFTY2 |
| RPLP0 | UHRF1 | | MORC4 | | EGFR |
| TXNIP | LIMS1 | | CHEK1 | | HOXC11 |
| ALMS1 | AASS | | SYDE1 | | HAS2 |
| CTTNBP2 | CDK4 | | BTN2A2 | | HOXA9 |
| EBF4 | DHX40 | | FOXM1 | | FCGBP |
| LOXL3 | GUSB | | MEST | | MSR1 |
| GGA1 | VEGFA | | FANCC | | CLEC4E |
| SALL1 | MCM7 | | ESCO2 | | COL4A2 |
| HLA-B | CDK6 | | FLNA | | NEK2 |
| PABPC1 | MUC1 | | PTBP1 | | RPE65 |
| SETDB1 | HLA-DPB1 | | DBF4 | | SERINC2 |
| IRF9 | SCIN | | CDK1 | | AQP1 |
| SOX8 | COL22A1 | | ATAD2 | | ERCC6L |
| PTBP1 | AVIL | | TNC | | CD44 |
| NBPF10 | CALD1 | | MCM5 | | HOXC5 |
| SLC12A4 | PDIA4 | | CRISPLD1 | | IQGAP3 |
| CHD1L | JAG1 | | ZNF20 | | CENPI |
| CAD | ZNF217 | | CKS2 | | C7orf57 |
| KAT2A | LOXL3 | | KIFC1 | | NES |
| NES | NFE2L3 | | CDC45 | | COL1A2 |
| SARM1 | AURKA | | CASP2 | | NID1 |
| BMP7 | BRCA1 | | ELN | | FANCD2 |
| MIF4GD | USP49 | | CDK4 | | MEST |
| TBX2 | ITGB2 | | STK17A | | TACC3 |
| UNC13D | CDCA7L | | NUP107 | | VSIG4 |
| MAN2B1 | ZFP36L2 | | SLFN12 | | HOXA13 |
| FANCE | TEAD4 | | CPVL | | BRIP1 |
| GFAP | TNFRSF10B | | TRIP10 | | GATA4 |
| MCL1 | HOXA5 | | KANK2 | | MACC1 |
| C19orf57 | ZFP36 | | NRM | | PYGL |
| NOTCH1 | MRC2 | | CTNNAL1 | | ABCA13 |
| C1QTNF5 | C1R | | STK38 | | HOXA6 |
| CHD4 | PSMC3IP | | GAL3ST4 | | TEAD2 |
| PYCR1 | SPAG5 | | RNASEH2A | | FANCI |
| PLCB3 | ICAM1 | | ST14 | | ITPRIPL1 |
| DVL2 | CD74 | | HELLS | | CPVL |
| BCAN | CLEC5A | | EZH2 | | CBX2 |
| ITGAX | LAMA5 | | ABCB4 | | CD248 |
| UBA7 | HLA-A | | PRIM2 | | F2RL3 |
|  | UNC5CL | | NUP205 | | TAGLN2 |
|  | MAN2B1 | | MCM10 | | S100A8 |
|  | LAMB4 | | PSMB8 | | CYP27B1 |
|  | CD72 | | PKMYT1 | | PLEKHG2 |
|  | HLA-B | | HNF4G | | SMO |
|  | SAMD9 | | HLA-A | | COL22A1 |
|  | SLC25A13 | | RAI14 | | COL5A1 |
|  | LAMB1 | | GNAI3 | | FKBP10 |
|  | TRAIP | | SLC4A2 | | F13A1 |
|  | NUP205 | | ABCB7 | | CARD16 |
|  | PCNA | | HAUS1 | | BST1 |
|  | TRIM56 | | CARD6 | | BEST3 |
|  | FAM83D | | IFI16 | | SOX4 |
|  | GPC2 | | MAN2B1 | | S1PR3 |
|  | C1orf226 | | EPHB4 | | ERP27 |
|  | CLCF1 | | SMO | | LAMP3 |
|  | PSMB8 | | TGFB1I1 | | MYO1G |
|  | CPXM1 | | DOCK11 | | EDARADD |
|  | PTPRZ1 | | AFAP1L1 | | ELN |
|  | TAP1 | | TSPAN6 | | LIMA1 |
|  | PILRA | | ZNF311 | | CASP1 |
|  | LMNA | | MATN2 | | SLAMF7 |
|  | AHR | | TTC23 | | CD1D |
|  | LRIG3 | | MAP7D3 | | CD70 |
|  | CACNA2D4 | | PITX1 | | PRAME |
|  | ITGAX | | ATP11C | | VEGFA |
|  | TAGLN2 | | ZNF486 | | HSPA6 |
|  | TRIM24 | | FBXO43 | | SLAMF8 |
|  | DCAF13 | | TRIM24 | | FOLR1 |
|  | AURKB | | PROCR | | SLC7A7 |
|  | RECQL4 | | BTN3A2 | | RAD54L |
|  | TCIRG1 | | RDX | | C1QTNF1 |
|  | DNMT3B | | LPCAT3 | | RBBP8 |
|  | CKAP2L | | NES | | HSPG2 |
|  | EPHB4 | | PTK7 | | PLBD1 |
|  | CCDC136 | | STXBP4 | | GIMAP2 |
|  | S100A4 | | A2M | | PTK7 |
|  | PARP12 | | LEF1 | | AEBP1 |
|  | SIGLEC1 | | MUC1 | | CDCP1 |
|  | HDAC7 | | C1QTNF1 | | CTHRC1 |
|  | CSPG4 | | AR | | LFNG |
|  | CLEC12A | | TMX1 | | COL28A1 |
|  | TCF7L1 | | IKBKB | | ECM2 |
|  | MICALL2 | | LRRCC1 | | SH2D4A |
|  | NOTCH1 | | ZNF69 | | EVC2 |
|  | UNC13D | | MSI1 | | DMBX1 |
|  | COL18A1 | | RNF122 | | GAL3ST4 |
|  | PLVAP | | NPL | | SIGLEC1 |
|  | CCDC142 | | INPPL1 | | TIMELESS |
|  | EME1 | | PLEKHG2 | | LY96 |
|  | LOXL2 | | PIF1 | | XPNPEP2 |
|  | SBNO2 | | GART | | DBF4 |
|  | ALMS1 | | MCM9 | | IL7 |
|  | CNN2 | | NBPF9 | | RPLP0 |
|  | LAMB2 | | LYAR | | JAG1 |
|  | CD84 | | CCDC102B | | FLNA |
|  | MTBP | | PHF19 | | ANKRD53 |
|  | HIST1H3F | | KIAA1958 | | PXDN |
|  | ZNF479 | | DCAF13 | | ABCB4 |
|  | BTN2A2 | | SOX13 | | CTSS |
|  | KIF7 | |  | | IL1RAP |
|  | PDGFRB | |  | | RAB38 |
|  | TRIM47 | |  | | SDC1 |
|  | DST | |  | | EPHB4 |
|  | EMILIN1 | |  | | FNDC3B |
|  | CHTF18 | |  | | C1QC |
|  | MBTD1 | |  | | CHEK1 |
|  | C15orf39 | |  | | KCNE3 |
|  | SMO | |  | | SAMD9 |
|  | COL6A1 | |  | | DNALI1 |
|  | RRBP1 | |  | | NAMPT |
|  | DOCK6 | |  | | MYC |
|  | NT5DC2 | |  | | STC1 |
|  | UBA7 | |  | | MFRP |
|  | HK3 | |  | | OAS1 |
|  | MATN2 | |  | | FBP1 |
|  | DMTF1 | |  | | TES |
|  | LAG3 | |  | | CALD1 |
|  | VEZF1 | |  | | MSN |
|  | ZFHX4 | |  | | RAD54B |
|  | MST1 | |  | | IFI16 |
|  | TMC6 | |  | | COL14A1 |
|  | RBM28 | |  | | MTTP |
|  | PLEKHA8 | |  | | SLAMF6 |
|  | GPR84 | |  | | STEAP1 |
|  | HIST1H1E | |  | | VAMP5 |
|  | REST | |  | | TPRG1 |
|  | PTPN12 | |  | | PRSS23 |
|  | NCAPD2 | |  | | CD300A |
|  | CD68 | |  | | BCHE |
|  | DDR1 | |  | | PTPRZ1 |
|  | BTN3A1 | |  | | LAMB1 |
|  | ARPC1B | |  | | C1orf112 |
|  | | CDK5RAP3 | | LAMB4 | |
|  | CCDC93 | |  | | CD207 |
|  | ZNF3 | |  | | TRIP6 |
|  | ENO3 | |  | | RASEF |
|  | SULF2 | |  | | FAM20A |
|  | SHMT2 | |  | | PTBP1 |
|  | | RAD51AP1 | | FCER1A | |
|  | GRN | |  | | SAMD9L |
|  | SMC6 | |  | | CABP4 |
|  | CCDC146 | |  | | C1S |
|  | ABCB4 | |  | | DOK2 |
|  | PLIN3 | |  | | GPR141 |
|  | HK2 | |  | | UPP1 |
|  | PMM2 | |  | | ABCA1 |
|  | PEX1 | |  | | SPAG4 |
|  | TPST1 | |  | | CASZ1 |
|  | TRAF4 | |  | | MBOAT1 |
|  | ZNF462 | |  | | NECAP2 |
|  | TRIP10 | |  | | PLTP |
|  | C19orf57 | |  | | IL32 |
|  | CCT6A | |  | | SERPING1 |
|  | RBM33 | |  | | RPSAP58 |
|  | RPN2 | |  | | MPZL2 |
|  | NPEPL1 | |  | | ADAMTS15 |
|  | SNX5 | |  | | ATAD2 |
|  | MYO9B | |  | | CTSW |
|  | A2M | |  | | APOBEC3G |
|  | SLC4A2 | |  | | ANKFN1 |
|  | RNF213 | |  | | BLM |
|  | MDFIC | |  | | CD63 |
|  | RHBDF1 | |  | | CLSPN |
|  | STAB1 | |  | | SMAGP |
|  | RGL2 | |  | | GLI2 |
|  | MYC | |  | | GPC2 |
|  | POLE | |  | | ATP6V0D2 |
|  | | CCDC102A | | ADAMTSL4 | |
|  | PAXIP1 | |  | | AVIL |
|  | MYO1F | |  | | GRHL3 |
|  | HLA-E | |  | | SLC28A1 |
|  | FANCE | |  | | CNN2 |
|  | BOC | |  | | SLC26A7 |
|  | MAPRE1 | |  | | PSTPIP2 |
|  | CAD | |  | | ZYX |
|  | ZNF439 | |  | | FAM177B |
|  | PLEKHH3 | |  | | MAN2B1 |
|  | ZNF823 | |  | | PRCP |
|  | KANK2 | |  | | IFI35 |
|  | LMNB2 | |  | | TNFSF8 |
|  | DEF6 | |  | | MCM7 |
|  | FGL2 | |  | | TP53INP1 |
|  | RASAL3 | |  | | SSR2 |
|  | CD300C | |  | | SLC39A1 |
|  | TGFB1I1 | |  | | TCF3 |
|  | POT1 | |  | | PSPH |
|  | ZNF783 | |  | | IL15RA |
|  | ABCC1 | |  | | EMILIN1 |
|  | DNAJC2 | |  | | RACGAP1 |
|  | NCAPG2 | |  | | CLECL1 |
|  | UBR5 | |  | | KCTD14 |
|  | HM13 | |  | | PCDH18 |
|  | FXYD5 | |  | | FLT4 |
|  | ZNF558 | |  | | TNFRSF1A |
|  | MACC1 | |  | | SLC16A3 |
|  | PGLS | |  | | METTL1 |
|  | BTG2 | |  | | C7orf31 |
|  | RPLP0 | |  | | PTGS1 |
|  | PLOD3 | |  | | TSKU |
|  | | HIST1H2BD | | NCF1 | |
|  | DNMT1 | |  | | MTMR11 |
|  | APBA3 | |  | | PIPOX |
|  | TMEM168 | |  | | NUAK2 |
|  | PKMYT1 | |  | | OLFML2B |
|  | | MAPKAPK2 | | STXBP4 | |
|  | LIX1L | |  | | NFE2L3 |
|  | C1orf56 | |  | | ACE |
|  | WDR62 | |  | | ELF4 |
|  |  | |  | | ECT2 |
|  |  | |  | | BRCA1 |
|  |  | |  | | ATP6V1C2 |
|  |  | |  | | TPST1 |
|  |  | |  | | A2M |
|  |  | |  | | PARP12 |
|  |  | |  | | LIPG |
|  |  | |  | | TRIM4 |
|  |  | |  | | OASL |
|  |  | |  | | RCN1 |
|  |  | |  | | TLR5 |
|  |  | |  | | RPL22L1 |
|  |  | |  | | GALK1 |
|  |  | |  | | NUP205 |
|  |  | |  | | RFC4 |
|  |  | |  | | GSTK1 |
|  |  | |  | | CD3E |
|  |  | |  | | CCDC138 |
|  |  | |  | | ENG |
|  |  | |  | | CDKN1A |
|  |  | |  | | SLC4A2 |
|  |  | |  | | FKBP9 |
|  |  | |  | | ARHGEF6 |
|  |  | |  | | CIITA |
|  |  | |  | | F11R |
|  |  | |  | | IL18RAP |
|  |  | |  | | PTPN12 |
|  |  | |  | | RASGRP4 |
|  |  | |  | | APOBEC3F |
|  |  | |  | | THBS3 |
|  |  | |  | | DCAF13 |
|  |  | |  | | MPZL1 |
|  |  | |  | | DDR2 |
|  |  | |  | | ZC3HAV1 |
|  |  | |  | | OAS2 |
|  |  | |  | | TFEC |
|  |  | |  | | TMEM67 |
|  |  | |  | | VAT1 |
|  |  | |  | | LAPTM4A |
|  |  | |  | | MCM6 |
|  |  | |  | | NUP107 |
|  |  | |  | | NCAPG2 |
|  |  | |  | | CUBN |
|  |  | |  | | CLEC1A |
|  |  | |  | | GEM |
|  |  | |  | | WDR62 |
|  |  | |  | | TRIM14 |
|  |  | |  | | CREB3L4 |
|  |  | |  | | PIH1D2 |
|  |  | |  | | RCN3 |
|  |  | |  | | PRIM1 |
|  |  | |  | | SPAG5 |
|  |  | |  | | DENND2A |
|  |  | |  | | APH1A |
|  |  | |  | | LY9 |
|  |  | |  | | FHL3 |
|  |  | |  | | EFNB1 |
|  |  | |  | | CCDC81 |
|  |  | |  | | ATP8B3 |
|  |  | |  | | LIN9 |
|  |  | |  | | SUMF2 |
|  |  | |  | | C3 |
|  |  | |  | | TGFBR1 |
|  |  | |  | | SCARF2 |
|  |  | |  | | ALG3 |
|  |  | |  | | PILRA |
|  |  | |  | | CC2D2A |
|  |  | |  | | HRCT1 |
|  |  | |  | | KLF17 |
|  |  | |  | | ZDHHC4 |
|  |  | |  | | IQUB |
|  |  | |  | | DHX40 |
|  |  | |  | | NCKAP1L |
|  |  | |  | | C12orf60 |
|  |  | |  | | CDA |
|  |  | |  | | RECQL |
|  |  | |  | | ORAI1 |
|  |  | |  | | GNB4 |
|  |  | |  | | NAALADL1 |
|  |  | |  | | MRPL13 |
|  |  | |  | | CBX8 |
|  |  | |  | | MAGEA12 |
|  |  | |  | | LPP |
|  |  | |  | | GORAB |
|  |  | |  | | HCK |
|  |  | |  | | NOTCH3 |
|  |  | |  | | MAP4K1 |
|  |  | |  | | FZD1 |
|  |  | |  | | CD37 |
|  |  | |  | | GZMM |
|  |  | |  | | TCTN2 |
|  |  | |  | | GPN1 |
|  |  | |  | | CEP135 |
|  |  | |  | | HMBS |
|  |  | |  | | NUBP1 |
|  |  | |  | | CCNE2 |
|  |  | |  | | MMP19 |
|  |  | |  | | ETS1 |
|  |  | |  | | IQCG |
|  |  | |  | | TRIM24 |
|  |  | |  | | CD300C |
|  |  | |  | | RAVER1 |
|  |  | |  | | DDX11 |
|  |  | |  | | SNRPB |
|  |  | |  | | ZNF683 |
|  |  | |  | | ZC3H12A |
|  |  | |  | | MNDA |
|  |  | |  | | RBM38 |
|  |  | |  | | SPAG1 |
|  |  | |  | | ABCD1 |
|  |  | |  | | GALNT1 |
|  |  | |  | | MKS1 |
|  |  | |  | | KIF24 |
|  |  | |  | | VSIG10 |
|  |  | |  | | NOD1 |
|  |  | |  | | APBA3 |
|  |  | |  | | SH2B2 |
|  |  | |  | | RIPK1 |
|  |  | |  | | NLRC4 |
|  |  | |  | | ABHD3 |
|  |  | |  | | ZMYM1 |
|  |  | |  | | SKP2 |
|  |  | |  | | ITGB8 |
|  |  | |  | | STXBP2 |
|  |  | |  | | RFX2 |
|  |  | |  | | SEC24D |
|  |  | |  | | VWF |
|  |  | |  | | ATP11C |
|  |  | |  | | PKN3 |
|  |  | |  | | LAT2 |
|  |  | |  | | ZNF3 |
|  |  | |  | | RPS4X |
|  |  | |  | | RMI1 |
|  |  | |  | | RREB1 |
|  |  | |  | | TBXAS1 |
|  |  | |  | | LRRCC1 |
|  |  | |  | | TBC1D10C |
|  |  | |  | | TTC27 |
|  |  | |  | | CHCHD3 |
|  |  | |  | | ITGA7 |
|  |  | |  | | HEXA |
|  |  | |  | | TNFRSF4 |
|  |  | |  | | SRGAP1 |
|  |  | |  | | ROBO4 |
|  |  | |  | | MCL1 |
|  |  | |  | | EIF3E |
|  |  | |  | | ZNF599 |
|  |  | |  | | POLA2 |
|  |  | |  | | DOCK6 |
|  |  | |  | | DISC1 |
|  |  | |  | | APOBEC3H |
|  |  | |  | | TMEM182 |
|  |  | |  | | CLEC7A |
|  |  | |  | | UACA |
|  |  | |  | | IRAK3 |
|  |  | |  | | GIMAP4 |
|  |  | |  | | LAMC3 |
|  |  | |  | | KDR |
|  |  | |  | | AP4M1 |
|  |  | |  | | CYP2U1 |
|  |  | |  | | 44531 |
|  |  | |  | | LPXN |
|  |  | |  | | SOX13 |
|  |  | |  | | RSU1 |
|  |  | |  | | REST |
|  |  | |  | | ACVRL1 |
|  |  | |  | | ZAP70 |
|  |  | |  | | NAALADL2 |
|  |  | |  | | RPS21 |
|  |  | |  | | NONO |
|  |  | |  | | PXN |
|  |  | |  | | TRAF3IP3 |
|  |  | |  | | MIDN |
|  |  | |  | | IRAK1 |
|  |  | |  | | WIPI1 |
|  |  | |  | | EPS8 |
|  |  | |  | | IFRD1 |
|  |  | |  | | ZNF433 |
|  |  | |  | | NPHP1 |
|  |  | |  | | NME7 |
|  |  | |  | | NANP |
|  |  | |  | | DDX59 |
|  |  | |  | | ARHGAP9 |
|  |  | |  | | RPL11 |
|  |  | |  | | CLPP |
|  |  | |  | | STARD3NL |
|  |  | |  | | GIMAP6 |
|  |  | |  | | LMCD1 |
|  |  | |  | | HES1 |
|  |  | |  | | MBD6 |
|  |  | |  | | SOX9 |
|  |  | |  | | PHACTR4 |
|  |  | |  | | DARS |
|  |  | |  | | TMEM209 |
|  |  | |  | | SHKBP1 |
|  |  | |  | | TMEM79 |
|  |  | |  | | LAMA5 |
|  |  | |  | | NOL11 |
|  |  | |  | | MUTYH |
|  |  | |  | | AIDA |
|  |  | |  | | NVL |
|  |  | |  | | AKR1A1 |
|  |  | |  | | CTAGE1 |
|  |  | |  | | PAFAH2 |
|  |  | |  | | DKC1 |
|  |  | |  | | ADPGK |
|  |  | |  | | VEZF1 |
|  |  | |  | | RNF19A |
|  |  | |  | | NUP188 |
|  |  | |  | | CLDN15 |
|  |  | |  | | TMBIM4 |
|  |  | |  | | FBXL12 |
|  |  | |  | | MRPS12 |
|  |  | |  | | REPIN1 |
|  |  | |  | | SBNO2 |
|  |  | |  | | ERMAP |
|  |  | |  | | FAM136A |
|  |  | |  | | TCEA1 |
|  |  | |  | | CDC42EP4 |
|  |  | |  | | FBXO22 |
|  |  | |  | | RBMX |
|  |  | |  | | INPPL1 |
|  |  | |  | | MECOM |


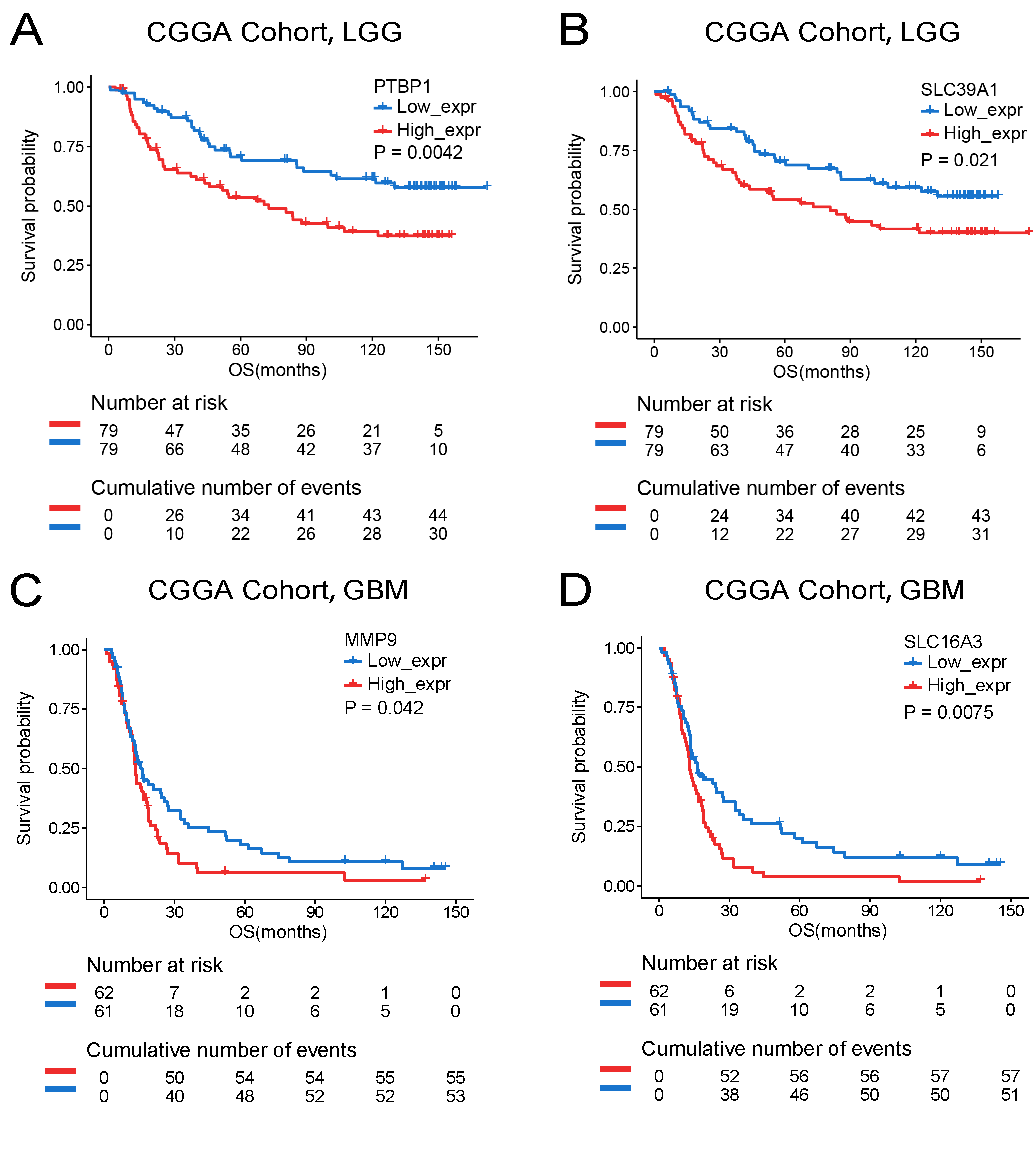


Fig S1. Identification of tumor antigens associated with the clinical outcome of LGG and GBM in CGGA cohort. A-D. Kaplan-Meier curves showing OS of LGG and GBM patients stratified on the basis of (A) PTBP1, (B) SLC39A1, (C) MMP9 and (D) SLC16A3 expression levels.


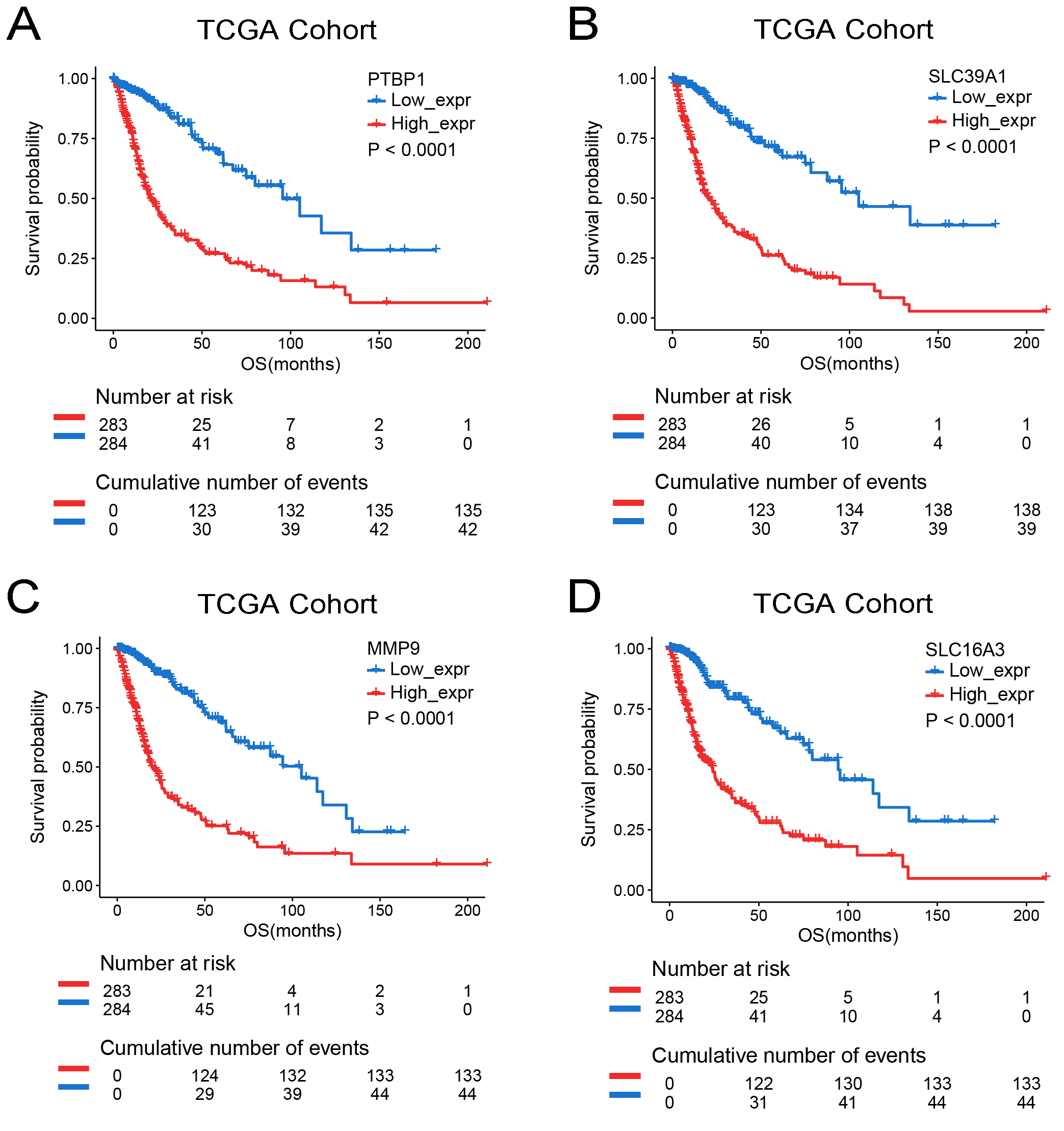


Fig S2. Identification of tumor antigens associated with the clinical outcome of glioma in TCGA cohort. A-D. Kaplan-Meier curves showing OS of glioma patients stratified on the basis of (A) PTBP1, (B) SLC39A1, (C) MMP9 and (D) SLC16A3 expression levels.


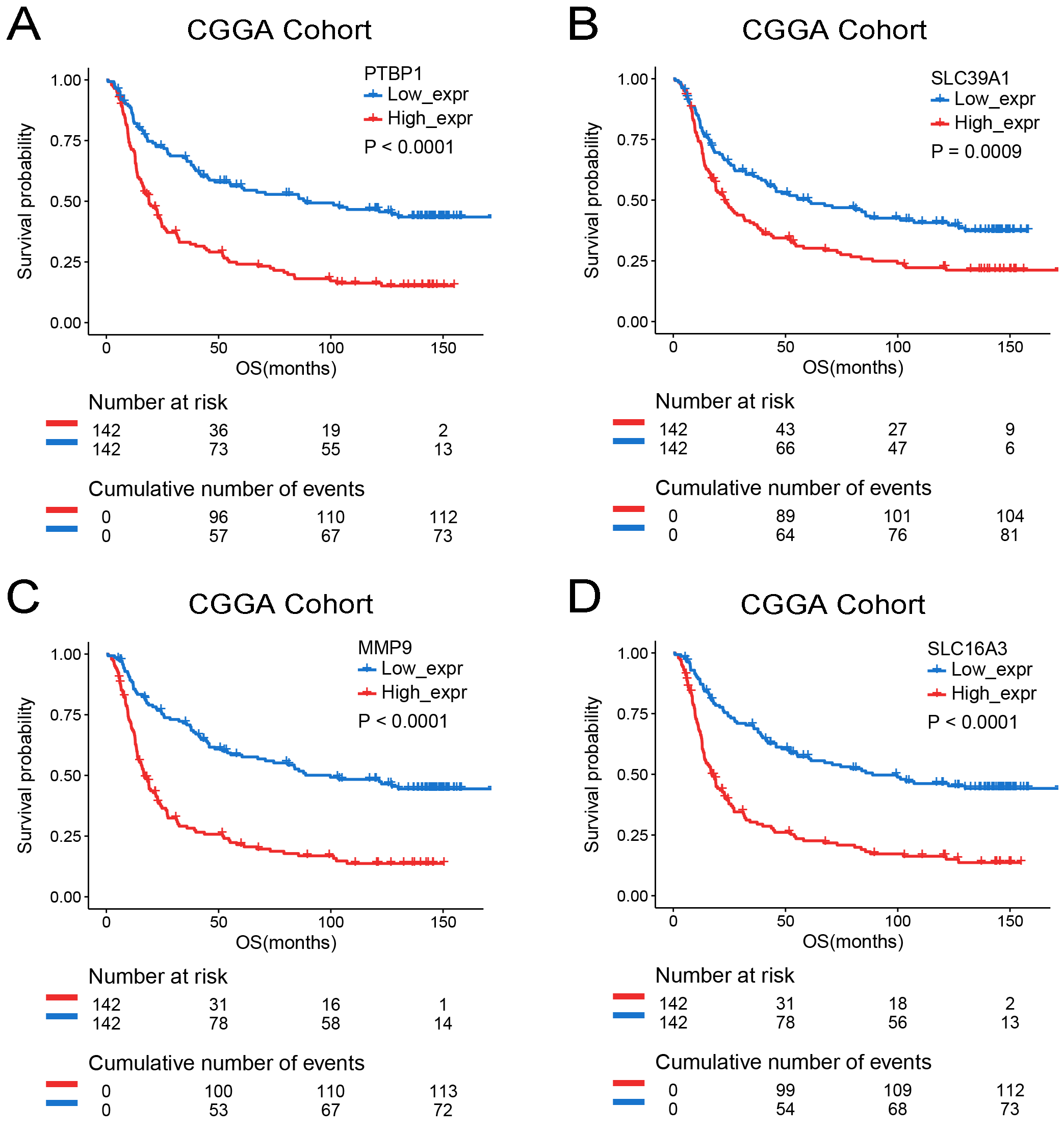


Fig S3. Identification of tumor antigens associated with the clinical outcome of glioma in CGGA cohort. A-D. Kaplan-Meier curves showing OS of glioma patients stratified on the basis of (A) PTBP1, (B) SLC39A1, (C) MMP9 and (D) SLC16A3 expression levels.


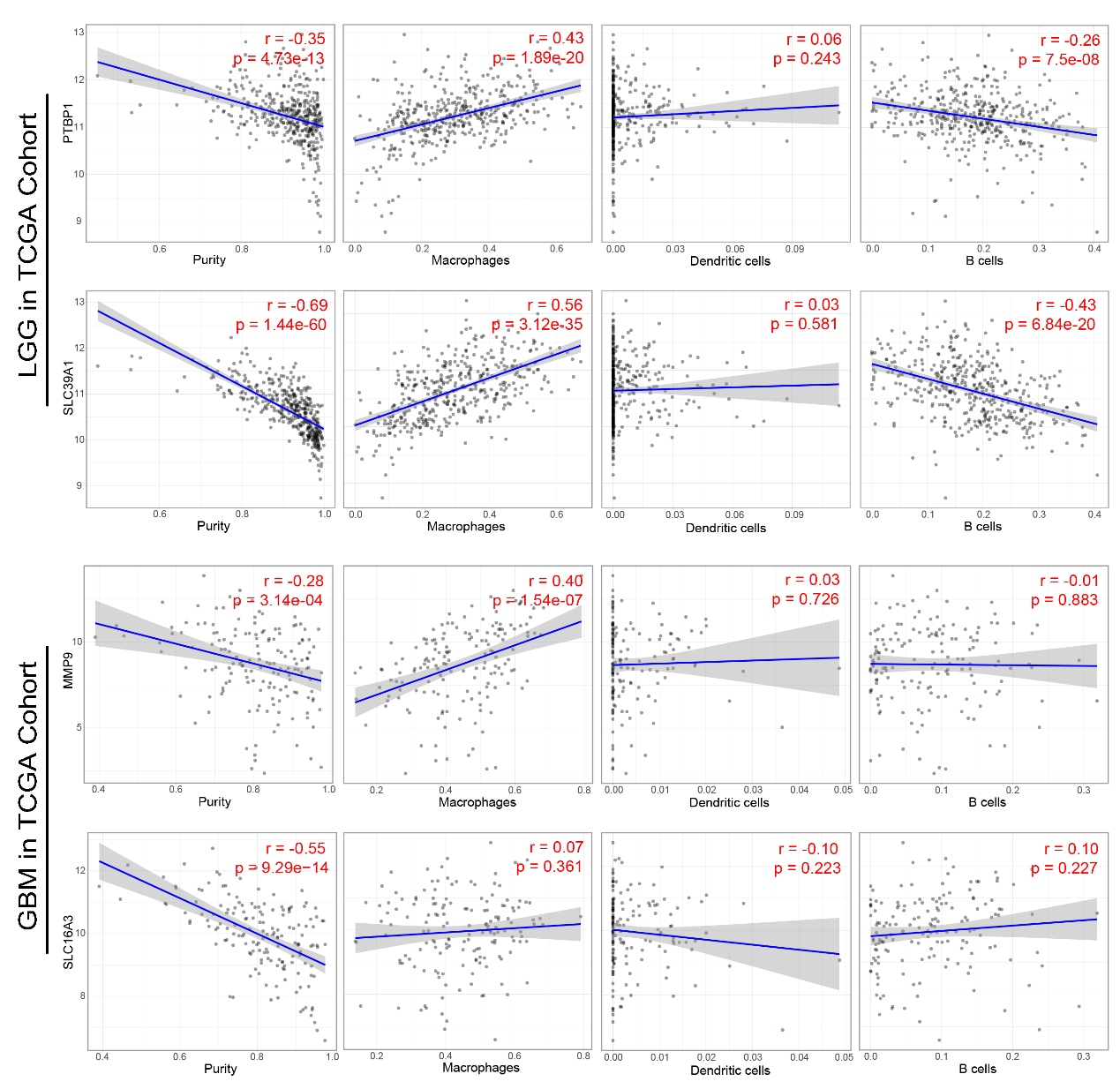
 Fig S4. Identification of tumor antigens associated with APCs in TCGA cohort. Correlation between the expression levels of(A) PTBP1, (B) SLC39A1, (C) MMP9 and (D) SLC16A3 and infiltration of macrophages, dendritic cells and B cells in LGG and GBM.


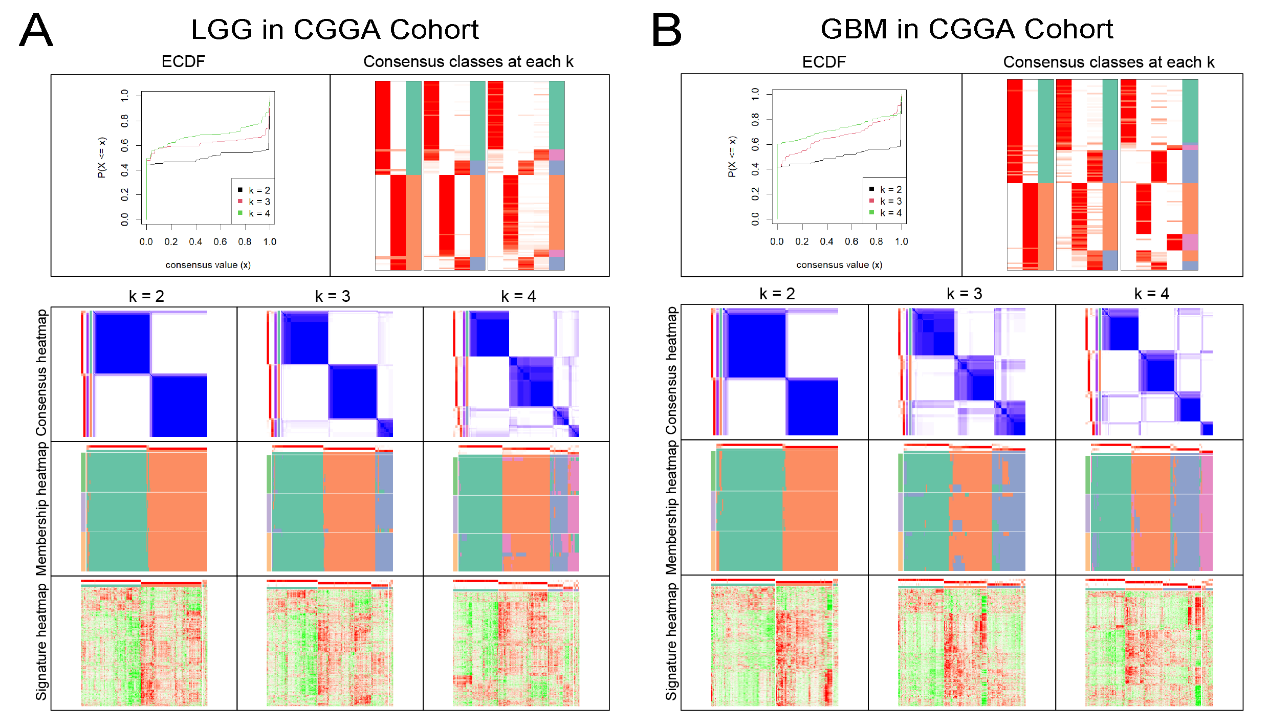


Fig.S5 Consensus clustering of LGG and GBM samples in CGGA cohort. (A-B) Color-coded heatmaps corresponding to the consensus matrices for k=2 to k=4 in LGG and GBM patients.


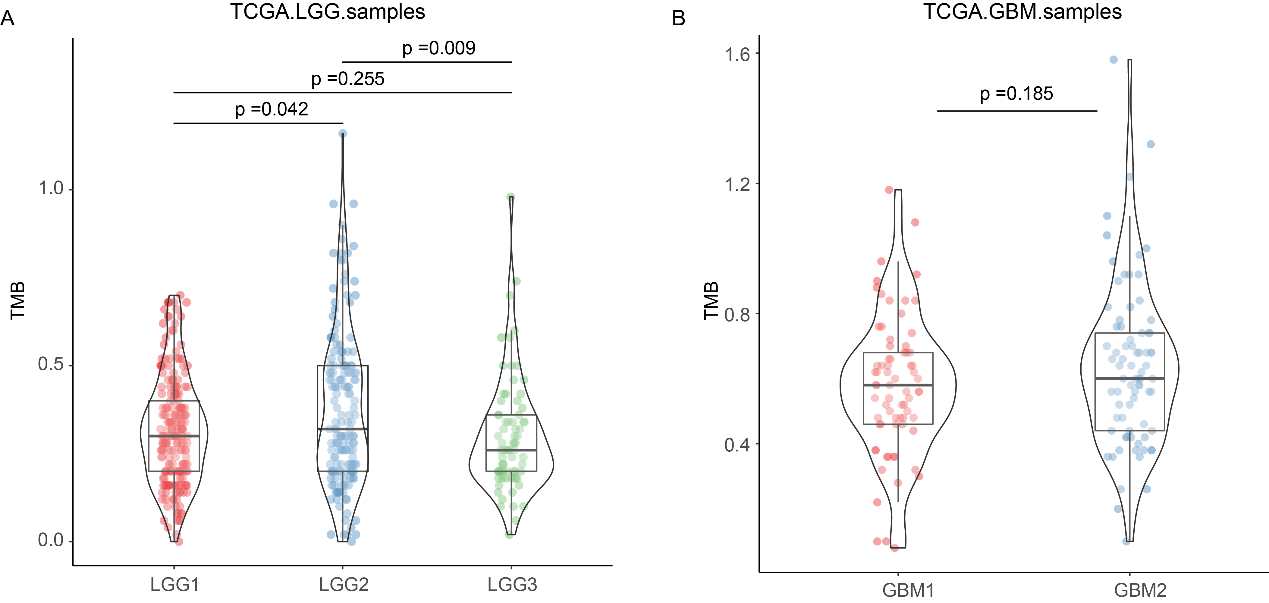


Fig.S6 Association of immune subtypes with TMB. A,B Tumor mutational burden (TMB) of different immune subtypes in LGG and GBM in TCGA cohort (Student’s t-test).
